# Fluidic innervation sensorizes structures from a single build material

**DOI:** 10.1126/sciadv.abq4385

**Published:** 2022-08-10

**Authors:** Ryan L. Truby, Lillian Chin, Annan Zhang, Daniela Rus

**Affiliations:** ^1^ Computer Science and Artificial Intelligence Laboratory, Massachusetts Institute of Technology, Cambridge, MA 02139, USA.; ^2^ Departments of Materials Science and Engineering and Mechanical Engineering, Northwestern University, Evanston, IL 60208, USA.

## Abstract

Multifunctional materials with distributed sensing and programmed mechanical properties are required for myriad emerging technologies. However, current fabrication techniques constrain these materials’ design and sensing capabilities. We address these needs with a method for sensorizing architected materials through fluidic innervation, where distributed networks of empty, air-filled channels are directly embedded within an architected material’s sparse geometry. By measuring pressure changes within these channels, we receive feedback regarding material deformation. Thus, this technique allows for three-dimensional printing of sensorized structures from a single material. With this strategy, we fabricate sensorized soft robotic actuators on the basis of handed shearing auxetics and accurately predict their kinematics from the sensors’ proprioceptive feedback using supervised learning. Our strategy for facilitating structural, sensing, and actuation capabilities through control of form alone simplifies sensorized material design for applications spanning wearables, smart structures, and robotics.

## Teaser

Architected materials are innervated with air-filled channels, integrating programmed mechanical behavior, sensing, and actuation.

## INTRODUCTION

Multifunctionality is a defining feature in the composition and forms of biological systems (*1*, *2*). For example, the xylem of vascular plants participates in water and nutrient transport while directly contributing to structural integrity and resiliency (*3*). Likewise, the hierarchical structure of skeletal muscles facilitates the innervation and vascularization of densely packed muscle fibers, fulfilling the actuation, proprioception, and metabolic needs of vertebrate locomotion (*4*, *5*). These materials and structures have evolved to address multiple needs in a single composite, enabling living organisms to efficiently achieve the performance required for their continued survival and evolutionary fitness. Inspired by these lessons, engineers have increasingly explored multifunctionality in materials design as a strategy to improve the performance, range of capabilities, and efficiency of a broad range of new technologies (*2*, *6*).

In particular, a large subset of emerging technologies require multifunctional materials with programmable mechanical properties and distributed sensing (*6*–*9*). Recent works have demonstrated that bioinspired somatosensory materials can potentially address key performance challenges in next-generation smart structures (*10*–*14*), wearable devices (*15*–*18*), and robots (*19*–*24*). However, the materials used in these applications typically have strict mechanical requirements, such as high strength-to-weight ratios, extreme stiffness or compliance, and stretchability. These constraints make it difficult to imbue existing optimized materials with sensing. Current approaches to creating sensorized materials—and multifunctional materials in general—involve the integration of multiple materials, either through manual assembly (*10*, *14*, *16*, *18*, *19*, *21*–*24*), microfabrication (*15*, *17*, *20*), or specialized three-dimensional (3D) printing methods (*11*–*13*). These fabrication techniques involve specialized, low-throughput, and/or complex methods or equipment that are often limited in the materials they can assemble. Their limitations prevent both the desired mechanical and sensory needs from being met optimally.

**Fig. 1.**
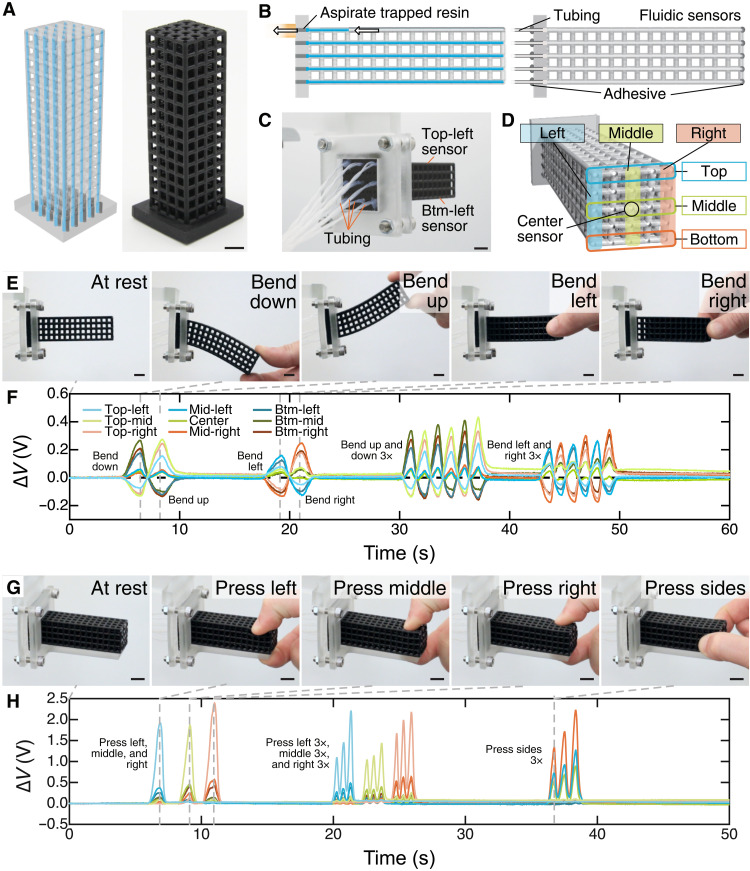
Fluidically innervated architected materials. (**A**) A render (left) and photograph (right) of a sensorized cubic lattice. (**B**) Trapped resin (blue) is aspirated from the printed networks via vacuum (left), and tubing is inserted into the fluidic channels after the final resin curing step (right). (**C**) The photograph shows the sensorized lattice from behind with tubing running from each sensor. (**D**) The schematic shows a sensor map for the nine sensors in the lattice. (**E**) Photographs from movie S2 during manual bending with (**F**) the corresponding voltage change, Δ*V*, over time from all sensors. (**G**) Photographs from movie S2 during tactile, pressing interactions with (**H**) the corresponding Δ*V* over time for the same sensors. Scale bars, 1 cm. Mid, middle; Btm, bottom.

Motivated by these challenges, we present a strategy for fabricating multifunctional materials with programmable mechanical behaviors and distributed sensing capabilities by controlling the form of a single build material. Our strategy involves the sensorization of architected materials with open fluidic networks, which we construct via 3D printing (Fig. 1) (*25*). Architected materials are a class of materials that achieve tailorable mechanical properties entirely via geometry (*26*). While this makes them excellent for achieving optimally programmed mechanical performance, architected materials’ dependence on geometry makes them difficult to sensorize. We surmount this problem by embedding empty, air-filled fluidic networks directly into an architected material’s internal structure. Once sealed, the networks’ internal pressures can be measured as voltage signals during deformation and can be used as sensory feedback. Similar cavity-based fluidic sensors have been developed for tactile feedback in robotic grippers with compliant fingertips (*27*, *28*) and soft robotic tactile skins (*29*). However, all of these methods rely on molding-based fabrication methods, yielding relatively large sensors that only provide tactile feedback. In contrast, our sensors are cocreated with the structure during 3D printing, enabling our sensors to be incorporated within more complex geometries and for more internal measurements. Likewise, the analog electronic feedback that we receive from our sensors is distinct from other types of soft fluidic sensors that provide solely binary mechanical feedback (*30*, *31*). For example, fluidic bistable valves have enabled an electronics-free approach to reflexive tactile sensing in soft robotic grippers that autonomously grasp upon contact with an object (*30*) and untethered soft robots that autonomously reverse gait upon activation of the fluidic sensor with an obstruction such as a wall (*31*). Sensing feedback from these fluidic sensors are only compatible with fluidic logic-based controllers (*30*, *31*), while our sensors can interface with traditional electronic controllers through a pressure transducer.

Our methods present three key opportunities. First, fluidic innervation provides a straightforward route for placing, distributing, and fabricating sensors within the sparse geometries of architected materials. Second, our fluidic sensing strategy avoids the time-varying effects common to current soft sensors. Soft sensors based on conductive liquids, piezoresistive elastomers, and viscoelastic waveguides are susceptible to drift and hysteresis because of their underlying microstructures and/or physicochemical behaviors (*32*). Our fluidic sensing strategy sidesteps these issues by directly reading pressure changes of closed, deformable volumes patterned within the structure. Lastly, our methods enable the creation of soft robotic systems with true somatosensory capabilities by using machine learning to associate sensor feedback with deformation for proprioception. Building off our recent efforts to develop compliant materials for motorized soft robots (*33*–*35*), we use our methods to sensorize a range of structures, including a group of materials called handed shearing auxetics (HSAs) that our group has previously introduced (*33*). This combination of motors and fluidic sensing yields a soft robotic system with robust actuation and perception capabilities. Our system is not susceptible to failure by overpressurization or leakage as is common in fluidically actuated soft robots, allowing us to operate the device for extensive periods of time. We collect large sensorimotor datasets and develop a deep neural network to proprioceptively predict the multi–degree-of-freedom (DOF) actuator’s kinematics. Fluidic innervation of the HSAs’ complex, sparse geometry represents an embodiment of a multifunctional construct that enables integrated structural, sensing, and actuation capabilities achieved from a single build material.

## RESULTS

### 3D printing sensorized architected materials

We first demonstrate our approach by 3D printing sensorized versions of common lattice architectures (Fig. 1A). As outlined in Fig. 1B, lattices with distributed fluidic networks are 3D printed from photopolymer resins using digital light processing (DLP). Nonpolymerized resin trapped within the fluidic networks during the printing process is aspirated by vacuum (movie S1), and channels are flushed with solvent and left empty. After parts are completely cured, fluidic sensor networks are connected to differential pressure transducers via elastomeric tubing (Fig. 1C) and sealed with adhesives. The fluidic channels are therefore filled with air only, and we use the perceivable pressure changes of the closed volumes as the basis of our fluidic sensors. While DLP affords the patterning resolution required to create our complex structures, the overall green body strength and printing resin’s viscosity and pot life limit the overall dimensions of fluidic features that we can pattern. We investigate the fabricable sensor geometries for multiple printing resins and find that the dimensions of successfully fabricated fluidic networks are resin dependent (fig. S1; see Materials and Methods).

According to Boyle’s law, deformation of the fluidically innervated materials results in changes of the internal pressure of the networks, *P*, inversely proportional to volume changes (i.e., decreases via compression and increases via extension). *P* is measured using in-line differential pressure transducers, which report *P*-dependent voltages, *V*. We demonstrate these operating principles in Fig. 1 using the simple cubic lattice printed from an elastomeric resin (EPU 40, Carbon). The lattice has nine straight fluidic sensors running along its length (Fig. 1D). The “top” and “bottom” (Btm) sensors lie on the top and bottom row of struts in the beam, respectively. The “middle” (Mid) sensors lie in the center row of struts, at the approximate neutral plane of the beam during downward and upward bending. “Left,” “middle,” and “right” sensors fall in the leftmost, middle, and rightmost columns of struts. Thus, left and right sensors lie on opposite sides of the neutral plane when the beam undergoes leftward and rightward bends. As the cubic lattice undergoes manual bending (Fig. 1, E and F, and movie S2), we observe decreases in *P* for the sensors lying above the neutral plane (Δ*V* < 0), while sensors below it report an increase in *P* (Δ*V* > 0). Notably, middle sensors lying in the neutral plane only produce a small voltage increase, likely due to compression by the solid struts parallel to the direction of bending. In Fig. 1 (G and H) and movie S2, we explore tactile sensor response by pressing on individual columns of sensors. In all instances, we observe increases in *P* during compression (Δ*V* > 0), which are consistent with our results from manual bending. We also observe a depth-dependent response, with sensors closer to the contact point providing greater Δ*V* than those further from it. Importantly, in both sets of experiments, we observe clear, highly responsive feedback from all sensors during lattice deformation. Any instances where Δ*V* does not return to Δ*V* = 0 after deformation result from either the lack of precise motion in manual deformations or the intrinsic creep and stress relaxation of the proprietary viscoelastic resins (*35*). Over a 12-hour thermal drift study, we observed stable sensing (fig. S3 and Supplementary Text). Figures S4 and S5 provide details of the data in Fig. 1 (E to H).

### Mechanical characterization of sensorized lattices

**Fig. 2.**
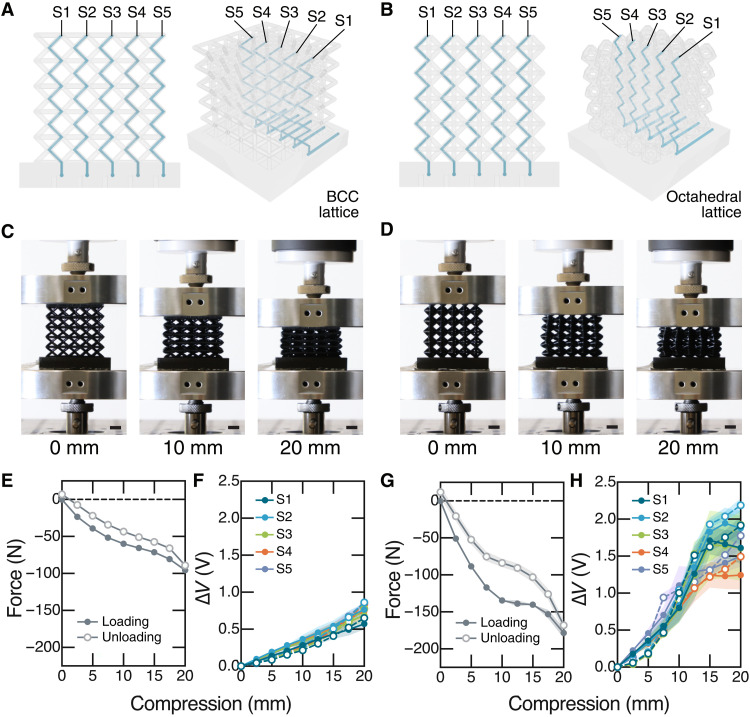
Sensory feedback in sensorized BCC and octahedral lattices. (**A**) BCC and (**B**) octahedral lattices sensorized with five fluidic sensors centered within the structure. (Left) Face-on, (right) perspective views. (**C**, **D**) Photographs from compression tests of sensorized BCC and octahedral lattices, respectively. The force versus compression data and voltage change, DV, versus compression response from all sensors are shown in (**E**, **F**) and (**G**, **H**) for the BCC and the octahedral lattices, respectively. In (E-H), data points and error bands represent mean and standard deviation (*n* = 3), and loading and unloading data are provided as filled and unfilled symbols, respectively. (Scale bars represent 1 cm).

We quantitatively study fluidic sensing with elastomeric, fluidically innervated body-centered cubic (BCC) and octahedral lattices undergoing compression (Fig. 2). Figure 2 (A and B) shows schematics of the BCC and octahedral lattices with five fluidic sensors of approximately equal volume (see table S1). We selected BCC (*M* = −13) and octahedral lattices (*M* = 0) for their similar architecture yet different bending- and stretching-dominated mechanical behaviors, respectively, according to Maxwell’s stability criterion, *M*. Figure S6 and movie S3 demonstrate the individual sensitivity of the sensors, named S1 to S5, through a tactile demonstration, where sensor responses are recorded from the octahedral lattice as the lattice is manually pressed directly onto or nearby the sensors.

During compression testing (Fig. 2, C and D), we observe that the octahedral lattice is stiffer than the BCC lattice (Fig. 2, E and G). This stiffness is reflected in the higher Δ*V* measured in the octahedral lattice’s sensors (Fig. 2, F and H), corresponding to higher forces required for compression. In these experiments, we observe that the five sensors in each lattice behave similarly for compressive forces below approximately 100 N. Above 100 N, sensors in the octahedral lattice show greater variability with increased compression on account of the lattices’ extreme, heterogeneous deformation in this regime.

**Fig. 3.**
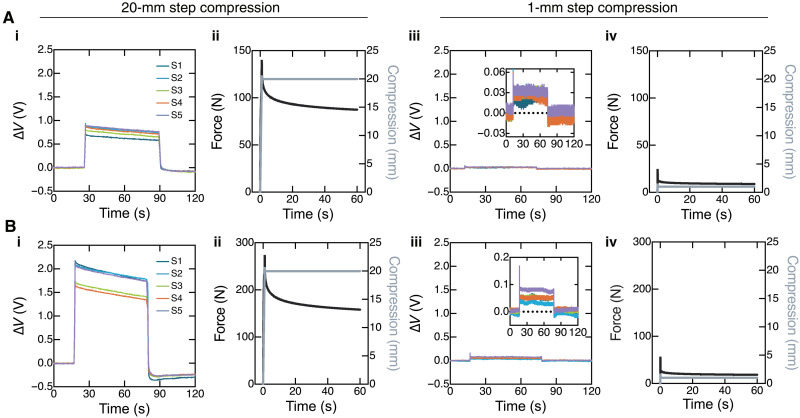
Characterizing fluidic sensor responses during step compressions. Sensor and mechanical responses are provided for sensorized (**A**) BCC and (**B**) octahedral lattices undergoing step compressions. Plots (i) and (iii) provide sensor voltage change, ∆*V*, over time for the five fluidic sensors, S1 to S5, during 20- and 1-mm step compressions, respectively. Plots (ii) and (iv) provide the corresponding force and compression data over time, as measured via Instron during 60-s step compressions of 20 and 1 mm, respectively. The insets in (A, iii) and (B, iii) are scaled to observe the small but measurable sensor changes in response to 1 mm of compression. ∆*V* = 0 is indicated in the insets by the dotted black line.

To understand the dynamic response of the fluidic sensors under step compressions, we recorded sensor responses for sensorized BCC and octahedral lattices compressed to fixed distances (Fig. 3 and fig. S7). Figure 3 provides the dynamic sensor responses of the BCC and octahedral lattices undergoing 60-s step compressions of 20 mm (Fig. 3, Ai and Bi) and 1 mm (Fig. 3, Aiii and Biii). The corresponding mechanical responses measured are provided in Fig. 3 (A, ii and iv, and B, ii and iv). The decrease in compressive force over the 60-s hold indicates stress relaxation in the lattices. For each lattice, we observe that the overall magnitude in sensor response Δ*V* decreases with decreasing compression distance. This is in agreement with Fig. 2. Even the 1-mm compressions produce a measurable Δ*V*, as shown in plots (iv) of Fig. 3. We also see that the magnitude in sensor response for each compression distance is greater for the stiffer octahedral lattice than the BCC. Importantly, though, we observe a time-varying decay in Δ*V* over the 60-s compression that corresponds to the stress relaxation response in Fig. 3 (A, ii and iv, and B, ii and iv). Movie S4 shows an example compression test at 20 mm with the octahedral lattice. In this video, the octahedral lattice requires several seconds to return to its initial dimensions when the compressive force is removed, revealing the extent of stress relaxation in these viscoelastic resins. Figure S7 shows additional sensor responses during step compressions at 5 and 10 mm.

Data from cyclic compression experiments in figs. S8 and S9 further reveal that the viscoelasticity of the cross-linked resins is responsible for any time-varying behavior in the sensors. Even after 10,000 cycles of compression (fig. S10), we observe largely nonhysteretic sensor responses given the fluidic sensing approach (Supplementary Text). Overall, the fluidic sensors’ performance during mechanical characterization suggests that our sensorization technique is practical for architected structures and is a reliable alternative to soft matter–based conductors.

### Sensorized HSAs

**Fig. 4.**
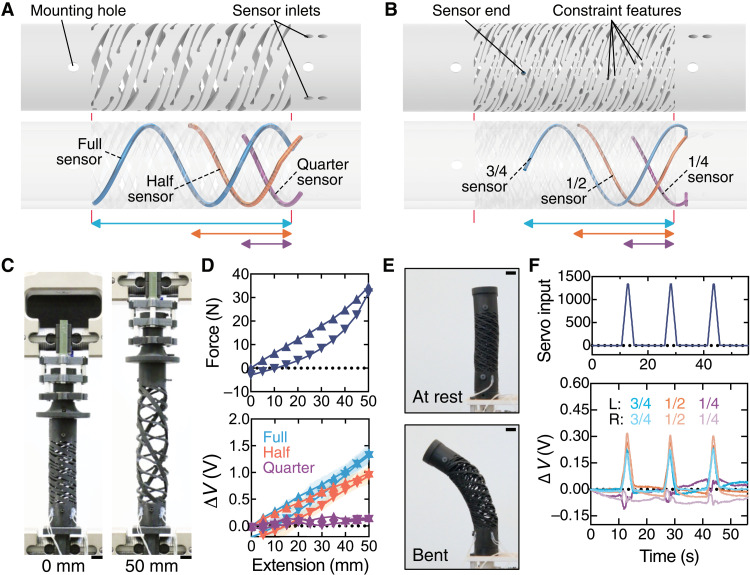
Sensorizing electrically actuatable HSAs. (**A** and **B**) Schematics of (A) straight and (B) bending sHSAs show the overall sHSA shape (top) and internal placement of the fluidic sensors (bottom). (**C**) The photographs show a straight sHSA at 0- and 50-mm extension. (**D**) Plots of extension force (top) and voltage change, Δ*V*, for the full, half, and quarter sensors (bottom) versus extension are provided for 1.5-mm sensor diameters. Error bands represent SD (*n* = 3). Triangles pointing upward and downward represent data points during extension from 0 to 50 mm and from 50 to 0 mm, respectively. (**E**) Photographs of a soft robotic actuator composed of two bending sHSAs of opposite handedness show at-rest and bent configurations. (**F**) Plots of servo input (top) and Δ*V* for the 3/4, 1/2, and 1/4 sensors in the left (L)– and right (R)–handed sHSAs (1-mm sensor diameters) (bottom) versus time are provided as the actuator undergoes three actuation cycles. Scale bars, 1 cm.

We next turn to the sensorization of HSAs, a recently developed class of architected materials that our group has developed for the design of motorized soft robots (*33*–*35*). Through a repeated joint linkage design, the HSA form tightly couples twisting with linear extension, enabling a single motor to drive a pair of HSAs as a compliant, soft robotic actuator. As with other architected materials, HSAs are difficult to sensorize because of their complex forms, and sensors must accommodate HSAs’ extreme deformation (*36*). Sensorizing via fluidic innervation bypasses this issue by allowing us to embed sensors within the HSA architecture. Using a flexible polyurethane resin (FPU 50, Carbon) (*35*), we 3D-printed two different sensorized HSAs (sHSAs), each with three embedded fluidic sensors. The first sHSA is based on a straight, unconstrained variety (Fig. 4A). The straight sHSAs’ “full,” “half,” and “quarter” sensors weave through the sHSA structure, terminating at 1×, 0.5×, and 0.25× the sHSA length, respectively. The second sHSA is based on a bending, constrained variety (Fig. 4B). Adding constraint features in the HSA turns otherwise linear extension into out-of-plane bending (*34*). The bending sHSAs’ sensors are called “1/4,” “3/4,” and “1/2.” We select these asymmetric sensor designs to sense different areas and modes of sHSA deformation. All investigated sHSA design parameters are provided in table S2.

We characterized fluidic sensor responses embedded in straight sHSAs via cyclic tensile extension (Fig. 4C). sHSA extension yielded increasing Δ*V* for the full and half sensors (with 1.5-mm diameters; Fig. 4D). As the sensors’ diameters were increased, we observed increasing sensitivity from the full and half sensors (fig. S11). This is expected because larger sensor volumes provide larger pressure changes during identical deformations (Supplementary Text and table S2). Similarly, the quarter sensor’s small volume leads to negligible sensitivity during linear extension in all cases. Following an analogous investigation, we found that a sensor diameter of 1 mm is appropriate for bending sHSAs given the reduced width of their widest struts (Fig. 4B). We then used two oppositely handed bending sHSAs and constructed the soft robotic finger shown in Fig. 4E, fig. S12, and movie S5. While the sensors in this device have the smallest volume of all sensors presented in this work, we see agreement between servo input and *V* for the 3/4 and 1/2 sensors in Fig. 4F. The small-volume 1/4 sensors report a noisy response because of their placement giving either a low sensitivity to bending or a minimal deformation with respect to the locations of the rigid constraint features.

### Learning kinematics of sHSA-based soft robots

**Fig. 5.**
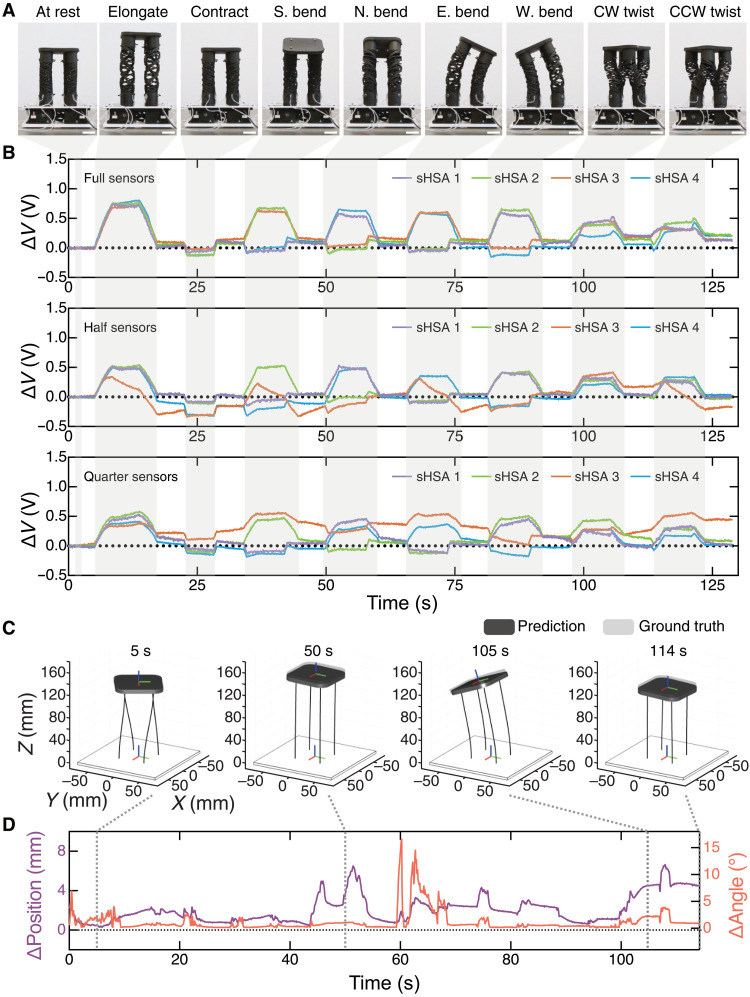
Proprioception in an sHSA robotic platform. (**A**) Photographs of an sHSA platform in motion and (**B**) corresponding changes in the voltage changes, Δ*V*, over time from the full (top), half (middle), and quarter sensors (bottom). (**C**) Snapshots from animations in movie S7 of the predicted and ground truth postures of the sHSA platform are provided for an actuation sequence from our test set with (**D**) a corresponding plot of the position (ΔPosition) and orientation (ΔAngle) error over time. The animation snapshots aid in visualizing the predicted and ground truth platform predictions at 5, 50, 105, and 114 s. Scale bars, 25 mm. S, south; N, north; E, east; W, west; CW, clockwise; CCW, counterclockwise.

To demonstrate a sensorized version of an electrically driven, sHSA-based soft robot, we constructed the system shown in Fig. 5A and fig. S13. The design of this four-DOF soft robotic platform was originally introduced in (*34*) and is composed here of four straight sHSAs with 1.5-mm-diameter full, half, and quarter sensors. Each sHSA is actuated by a different servo motor, with neighboring sHSAs having opposite handedness. Thus, the overall soft robot has 12 fluidic sensors and four servos for actuation. Figure 5 (A and B) and movie S6 show several of the maneuvers capable with the soft robot and the corresponding sensor responses, while fig. S14 provides the corresponding servo inputs.

Despite an increasing focus on data-driven approaches for solving longstanding challenges in soft robot control (*21*, *23*, *24*, *37*, *38*), the robustness and reliability of current soft robotic actuation and sensing strategies limit the size of datasets that can be acquired for proprioceptively determining shape or kinematics in soft sensorized robots. For example, popular pneumatically actuated soft robots are notoriously susceptible to premature failure from overpressurization with repeated actuation, making it challenging to build sufficiently large datasets. To evaluate the robustness of our system in alleviating performance challenges in data-driven approaches to soft robotic perception, we drove our soft robot through varying extents of its four DOFs for over 18 hours of sensorimotor experimentation, measuring sensor responses against a motion capture ground truth. This long data collection period is notably large for a soft robotic system. As described in the Materials and Methods, we then used this large, rich dataset to train a long short-term memory (LSTM)–based deep neural network capable of proprioceptively predicting the soft robot’s kinematics solely based on the data from 12 fluidic sensors as model inputs (fig. S16). Other data-driven sensing pipelines based on soft sensors with time-varying, hysteretic behaviors require neural networks with large numbers of hidden layers (*21*, *23*). We achieve accurate pose predictions of our sHSA platform with a simple network architecture. Figure 5 (C and D) and movie S7 show animated representations of our ground truth and model-predicted rigid body pose of the platform with corresponding Euclidean distance (i.e., position) error and rotational angle error over the course of representative time series (see fig. S18 for error explanation). While we certainly observe outlying instances, we generally observe that kinematic predictions align well with ground truth when soft robot motion is more continuous and holding of a pose is minimized. We suspect that this is due to the use of voltage changes as model inputs, as opposed to absolute voltage values. For interpreting the prediction data shown in Fig. 5 (C and D) and movie S7, we note that the rest length of the sHSA platform is 120 mm, and the maximum vertical extension is approximately 40 mm. The overall position error in Fig. 5D is small compared to these lengths. Thus, our motorized sHSAs and fluidic sensing strategies allow us to bring robust actuation and perception capabilities to soft robotics, bypassing both of these problematic issues for the field.

## DISCUSSION

We have demonstrated the versatility and integrity of our fluidic sensing strategy across a wide range of passive and active architected materials. Using a DLP-based printing method, we simultaneously create sensorized, architected materials and innervating vascular networks for fluidic sensing, bypassing the need for copatterning structural materials with conductive materials that have extreme time-varying behaviors. Importantly, following a bioinspired approach to multifunctional material design, our strategy of sensorizing structures through fluidic innervation greatly simplifies how materials with self-sensing and programmed mechanical behaviors can be fabricated. Through the sensorization of HSAs, we also introduce a soft robotic system representing the first demonstration of how a single build material can be architected to simultaneously achieve tailored structural properties, distributed sensing, and actuation capabilities. While many future sensorized materials and structures may require multiple materials for their design specifications, our method showcases a single-material approach to sensorizing architected materials, an important class of materials whose sparse, complex geometries complicate conventional approaches for fabricating sensing networks throughout. Moreover, many of these materials are already predominantly fabricated with DLP and other light-based printing methods, making our approach a strong fit for empowering future material research. Coupling the simplicity of our sensorization approach with other digitally fabricated materials could herald fundamentally new opportunities for the design and manufacturing of sensorized materials for wearable devices, smart structures, autonomous bioinspired robots, and beyond.

## MATERIALS AND METHODS

### 3D printing fluidically innervated architected materials

Fluidically innervated architected materials are parametrically designed in Rhino 6 and Grasshopper (Robert McNeel & Associates) with the Shortest Walk and Crystallon Grasshopper plugins. All parts are 3D-printed via DLP (Carbon M1 printer, Carbon Inc.) from commercially available elastomeric polyurethane (EPU 40), FPU 50, and transparent resin Loctite 3D IND405 (LOCTITE) photopolymer resins (all from Carbon Inc.). The sensorized lattices and sHSAs are printed from EPU and FPU, respectively. All transparent models are printed from Loctite 3D IND405. The resins and printed structures are used and postprocessed, respectively, according to the manufacturer’s protocols. Supports are printed for sHSAs only. Tables S1 and S2 provide design parameters for all sensorized lattices and sHSAs, respectively.

The fluidic networks must be emptied and cleared of residual resin to prevent occlusion of the sensors before any ultraviolet or thermal curing steps are taken in the postprocessing procedures. Upon removal of the part from the build platform, fluidic sensor networks are aspirated by vacuum. We use a vacuum to aspirate any remaining resin from printed fluidic networks because positive pressure can rupture the fluidic channels in the green body state. The channels are flushed with pressurized air, and isopropanol (IPA) is flushed into the inlet of each sensor for 5 to 15 s to verify that the channel is open. Channels are flushed a second time with pressurized air and IPA. Parts are dried with pressurized air and postprocessed according to the manufacturer’s specifications.

### Sensorization and readout electronics

Sensor networks are designed with an inlet and an outlet, where inlets are locations for sensor tubing and outlets are included to facilitate removal of residual resin. After postprocessing, the silicone tubing (outer diameter of 0.94 mm and inside diameter of 0.51 mm; McMaster Carr) is glued into each sensor inlet using Sil-Poxy (Smooth-On). Pressurized air is used to verify successful leak-proof adhesion of the tubing to the sHSA, and sensor outlets are closed by filling with a small amount of Sil-Poxy, creating a closed volume. Sensor tubing is then joined to differential pressure sensors by threading the sensor tubing into larger diameter tubing placed over the sensors’ ports. Three differential pressure sensors (Amphenol, All Sensors) are used to measure pressure changes in the ranges of 0 to 0.5 in H_2_O (0.5 INCH-D1-4V-MINI), 0 to 1 in H_2_O (1 INCH-D1-P4V-MINI), and 0 to 5 in H_2_O (5 INCH-D1-P4V-MINI). Range 0 to 5 in H_2_O differential pressure sensors is used for the fluidically innervated cubic, BCC, and octahedral lattices. Range 0 to 1 in H_2_O pressure sensors is used for the full and half sensors of straight sHSAs, as well as the 3/4 and 1/2 sensors of the bending sHSAs. Range 0 to 0.5 in H_2_O pressure sensors is used for the quarter and 1/4 sensors of the straight and bending sHSAs, respectively. All output voltage signals from differential pressure sensors are filtered by a low-pass filter (cutoff frequency of ≈30 Hz) and read by a digital acquisition unit (NI USB-6212 DAQ, National Instruments). All reported sensor outputs are unfiltered.

The differential pressure sensors have two ports. The nominal voltage at a zero differential pressure (i.e., when both ports are at atmospheric pressure) is 2.25 V (specification sheet, Miniature Amplified Low-Pressure Sensors, All Sensors). Therefore, one can select which port is connected to the fluidic sensor to achieve an increasing/decreasing voltage change with respect to 2.25 V for an increasing pressure in the fluidic sensor. As discussed in greater detail in Supplementary Text, we mitigate effects due to thermal drift by connecting the second port of the differential pressure sensor to a dummy line, which comprises identical silicone tubing that wraps around the tubing to the functional fluidic sensor. After all tubing are sealed and connected to the differential pressure sensor, we allow the sensors to reach equilibrium internal pressures for 48 hours, which is the time point on which a differential pressure sensor connected to a fluidic network reports a nominal voltage of approximately 2.25 V.

### Print parameter characterization

The pressure drop, Δ*P*, required to remove all remaining resin via vacuum is dependent on the length, *L*, and radius, *R*, of the network, per the Hagen-Poiseuille equation

$$\Delta P=\frac{8\mu L}{\pi R^{4}}Q$$

where μ is the resin viscosity and *Q* is the flow rate initiated by the vacuum. To evaluate the fabricability of fluidic networks in architected materials, cubic lattices innervated with straight fluidic channels are printed as model structures with which print parameters can be screened. The lattices have a unit cell size of 5 mm, width of five cells, length of five cells, and a strut diameter of 2 mm. Within each lattice, there are 21 straight fluidic channels that span the height of the lattice, corresponding to three replicates of seven different channel diameters (i.e., 2*R*) 0.5, 0.75, 1.0, 1.25, 1.5, 1.75, and 2.0 mm. Six different heights (i.e., *L*) are evaluated: 5, 10, 25, 50, 75, and 100 mm. Pressurized air is flushed through each channel, which are noted as successful if air flows through freely and failed if air flow is blocked by incomplete removal of resin.

### Characterization of sensorized lattices

Nine sensor responses in the cubic lattices are characterized by manual deformation in directional bending and pressing motifs. Five sensor responses from BCC and octahedral lattices are characterized using an Instron during cyclic, uniaxial compression. The BCC and octahedral lattices’ fluidic sensors have approximately equal volume and are positioned vertically within one central row of unit cells. Sensors from all sensorized lattices are measured at a sampling frequency of 50 Hz in all characterization experiments.

BCC and octahedral lattices undergo two rounds of break-in compression cycles before further characterization: 100 cycles of compression to 10 mm at 1 mm/s and 100 cycles of compression to 20 mm at 5 mm/s. The lattices are then compressed at 2.5-mm increments from 0 to 20 mm and then decompressed from 20 to 0 mm. At each (de)compression interval, a force measurement is recorded from the Instron, and the sensor readings are recorded for 10 s, over which a mean and SD output voltage is determined for each sensor. This process is repeated three times.

Step compressions on BCC and octahedral lattices are conducted on an Instron. The lattices are compressed at 20 mm/s by 20, 10, 5, and 1 mm for 60 s. These compression distances correspond to compressive strains of 0.4, 0.2, 0.1, and 0.02, respectively, as the lattice structures are approximately 50 mm in height. Compressive load is removed at 20 mm/s. Sensor readings are taken before, during, and after the step compression.

Lastly, the BCC lattice was cyclically compressed 10,000 cycles to 10 mm at 1 mm/s. For this study, sensor readings were recorded at 5 Hz.

### Characterization of sHSAs

Sensor responses from straight sHSAs are characterized using an Instron during cyclic, uniaxial extension. Straight sHSAs with fluidic sensor diameters of 0.75, 1, 1.25, 1.5, and 1.75 mm were studied. Custom adapters are used to mount the straight sHSAs to the Instron to allow for free rotation of one end of the sHSA while fixing the other. Straight sHSAs are stretched in 5-mm increments from 0 to 50 mm and then back to 0 mm, with sensor readings recorded from each sensor by measuring the mean and SD of the reading over 10 s of sampling at 50 Hz. Experiments are repeated three times. Before all experiments, straight sHSAs are cyclically extended 50 mm at 10 mm/s for 100 cycles as break-in to remove any strain-softening behaviors due to the Mullins effect. sHSAs are then cycled at 1 mm/s for 10 cycles to validate that no leaks are present.

Bending sHSA assemblies are actuated using Dynamixel MX-28 servos (ROBOTIS) and are controlled via MATLAB (MathWorks). Two sHSAs, each of opposite handedness, are counterrotated against each other in a geared setup with a single servo. Sensor readings are collected at ≈15 Hz.

### sHSA soft robotic platform

Soft robotic fingers and multi-DOF platforms are constructed [as previously described in (*35*)] from sHSAs. Briefly, sHSA fingers and platforms are based on 2 × 1 and 2 × 2 assemblies of sHSAs with opposite handedness, respectively. Fingers are driven by one servo motor, while platforms are driven by four servo motors (i.e., one servo per sHSA). All soft robotic systems are actuated using Dynamixel MX-28 servos (ROBOTIS) and are controlled via MATLAB (MathWorks).

### Data collection with sHSA platform

Data for neural network prediction of the sHSA platform’s pose were taken by driving the platform through a series of motions while simultaneously recording sensor values against a motion capture ground truth. For all experiments, the sHSA platform is actuated through a sequence of motions, returning to its neutral position after each one. The motions are elongation, contraction, bend forward (or north bend), bend backward (or south bend), bend left (or west bend), bend right (or east bend), clockwise twist, and counter-clockwise twist. For each trial, all of the motions are performed, but the order in which they are performed is randomized.

During these experiments, output voltage signals from differential pressure sensors are recorded by a digital acquisition unit (NI USB-6212 DAQ, National Instruments) through MATLAB (MathWorks). MATLAB also directly records servo position feedback from Dynamixel MX-28 servos (ROBOTIS). Ground truth readings are recorded through rigid body motion tracking via Motive (OptiTrack). Data across software are synchronized via the interpolation of UNIX time stamps associated with each measurement, resulting in a final sampling frequency of 15 Hz. To normalize across trials, the initial sensor measurement for each trial is recorded as 0 V, so further sensor readings are reported as the pressure difference in the fluidic sensors.

To add variance across trials, a specific servo velocity, extent of motion range, and end-of-movement hold time are chosen from a preselected list of options. This causes each trial to be of different lengths, making it harder for the neural network to track spurious patterns. For example, a faster servo velocity will result in the sequence being completed in less time. The servo speeds studied were 5, 10, 20, and 40 rpm. The hold times studied were 0, 5, and 10 s. The range fractions studied were 25, 50, 75, and 100% of full DOF range. For each given velocity, range, and hold time, five trials were conducted.

In summary, 240 trials were autonomously recorded over a single 7-hour period. Nineteen of these trials did not have accurate synchronization between the motion capture and MATLAB recordings and were omitted. Nine more of these trials were found to have errors in the data concatenation process and were omitted as well. These experiments formed the dataset used for the neural network training, testing, and validation. A total of 11 hours of additional experiments was conducted to debug the data collection script, to break in and calibrate the sHSAs, and to take videos and photographs.

### Neural network design, training, and testing

#### Learning problem

To estimate the forward kinematics of the four-DOF sHSA platform, a neural network that predicts its pose (i.e., position and orientation) solely using analog voltage readings from the fluidic sensors as input is developed. Because time-dependent effects such as stress relaxation and creep are inherent to viscoelastic materials used for HSAs, the input-output relation is modeled with LSTM networks, a class of neural networks commonly used for learning-based proprioception in soft robotics because of their ability to capture temporal relations (*21*, *23*, *24*, *37*).

#### Data preprocessing

Of the 212 remaining time sequences, the last 32 are set aside as the test set. Because these test data are neither used for model training nor for model selection, evaluating the final LSTM on it provides an unbiased estimate of the system performance in deployment. The data from the remaining 180 experiments account for ≈85% of the total data and form the basis for the training and validation sets. Because the longest of these recordings consists of 3688 time steps, which is difficult to learn even for LSTMs, sequences of a more manageable length are extracted by sliding a window of length 64 over each recording (fig. S15). To simultaneously augment the dataset, the window is slid with a stride of 20. The first data point in each recording (zero voltage difference for input data; initial pose for output data) is duplicated until the length of the recording equals 64 plus a multiple of 20. In this way, a total of 14,528 sequences with length 64 are generated. A total of 1453 (≈10%) are split off randomly as a validation set to evaluate a particular choice of hyperparameters during the model selection process. Lastly, training and validation sets are shuffled, inputs are normalized to zero mean and unit variance, and outputs are normalized to zero mean.

#### Neural network architecture

The neural network reads in a time sequence of 12D vectors (sensor voltages) and outputs a time sequence of 7D vectors (platform pose) with equal length (fig. S16). The inputs are first passed through stacked LSTM layers, where the number of layers and the number of hidden and cell states are tunable hyperparameters. The output of the LSTM layers are then passed through a fully connected layer with equal input and output dimensions, followed by ReLU (rectified linear unit) activation. The data are passed through another fully connected layer that outputs a 7D vector. Lastly, a normalization layer is applied to the four values corresponding to the orientation prediction because an orientation is given by a 4D vector that represents the coefficients of a unit quaternion. To mitigate overfitting, the first fully connected layer and all LSTM layers are equipped with a dropout probability of 0.2.

Because the coefficients of unit quaternions can be interpreted as points on a 4D hypersphere, the distance between two quaternions are described by the geodesic distance on the hypersphere. Furthermore, by definition of quaternions, antipodal points on the hypersphere describe the same orientation in 3D Euclidean space. However, the simple mean squared error (MSE) is found to be sufficient in practice, although it neglects both the curvature of the hypersphere and the symmetry of quaternions. This choice is reasonable as long as training converges, because the Euclidean distance approximates the geodesic distance for quaternions close to each other. The loss between predicted and ground truth pose is therefore computed as the sum of the position MSE and the quaternion MSE, although additionally weighted by a hyperparameter that balances the relative importance of minimizing these errors.

#### Model selection

Starting around an initial configuration found by following the best practice guidelines for training LSTMs (*39*), a grid search over the following hyperparameters is performed: quaternion weight (10, 100, and 1000), initial learning rate (0.001, 0.005, and 0.01), number of LSTM layers (1, 2, and 3), and number of hidden and cell states (50, 100, 150, and 200). Implemented in PyTorch, all models are trained with the Adam optimizer and a batch size of 32 for 100 epochs. The learning rate scheduler halves the learning rate when the validation error does not improve for 20 epochs. To avoid overfitting, an early stopping rule observing the loss on the validation set is used. In other words, while training with a particular choice of hyperparameters, only the model that shows the lowest validation error across all 100 epochs is retained. To account for randomness during neural network training, the validation loss for a particular choice of hyperparameters is averaged over a total of three runs. Lastly, the model with the lowest validation error across all possible choices of hyperparameters is retrained for 1000 epochs. Out of three runs, the run with the lowest validation loss is kept as the best model for evaluation on the test set. The summary statistics of this hyperparameter search can be found in table S1 and fig. S17.
