# Supplementary material for: Fluidic innervation sensorizes structures from a single build material
Source: Sci Adv. 2022 Aug 10;8(31):eabq4385. doi: 10.1126/sciadv.abq4385 (PMC9365281; doi:10.1126/sciadv.abq4385)
Supplement: Supplementary file 1 — Supplementary Text Figs. S1 to S24 Tables S1 to S3 [file sciadv.abq4385_sm.pdf]

Science Advances
AAAS

Supplementary Materials for

Fluidic innervation sensorizes structures from a single build material

Ryan L. Truby *et al.*

Corresponding author: Ryan L. Truby, rtruby@northwestern.edu

Sci. Adv. **8**, eabq4385 (2022)
DOI: 10.1126/sciadv.abq4385

The PDF file includes:

Supplementary Text
Figs. S1 to S24
Tables S1 to S3
Legends for movies S1 to S8

Other Supplementary Material for this manuscript includes the following:

Movies S1 to S8

Supplementary Text

Thermal Drift

Like most sensors, our fluidic sensors are susceptible to thermal drift. The ideal gas law informs us that our sensors' output voltages -- which are dependent on pressure, P, within the fluidic sensor -- are also dependent on temperature, T:

$$Pv = nRT$$

where v is the fluidic sensor volume, n is the moles of gas within the sensor, and R is the ideal gas constant. Moreover, we also observe that our sensors are n-dependent as well. Our gas-permeable resins and the elastomeric tubing used to connect the fluidic sensors to the differential pressure sensors can enable environmentally sensitive changes in n, impacting sensor output. Thus, when working with our two-port differential pressure sensors, we mitigate effects of thermal drift by creating a ``dummy line" that connects to the second port of the differential pressure sensor (Fig. S2). The dummy line serves as a closed volume of tubing of approximately equal length to that used to connect the fluidic sensor to the first port of the differential pressure sensor. We wrap the dummy line around the tubing associated with the fluidic sensor in a braid-like fashion. This reduces any environmental influences on sensor output if we were to leave the second pressure sensor port exposed to the ambient atmosphere. The dummy line effectively equilibrates along with the fluidic sensor with which it is associated.

Influence of Resin Viscoelasticity on Fluidic Sensor Response

Characterizing fluidic sensor responses via cyclic compression reveals that the photopolymer resins are viscoelastic: the maximum voltage during sensorized lattice compression to 10 or 20 mm decreases with increasing cycle count, indicating a strain-induced weakening response (Figs. S8 and S9). After approximately 70 cycles, the cyclic voltage response's peaks begin to stabilize. We also compressed a sensorized BCC lattice for 10,000 cycles to 10mm at 1 mm/s (Figs. S10A,B). While we see a similar strain-induced weakening trend in this experiment, we also observe a creep response in the EPU resin when analyzing the load and compression response versus time (Fig. S10C). After thousands of cycles, we begin to observe the lattice not fully recovering from compression, and load is measured as 0 N with the lattice no longer pressing against the Instron platen. Other soft material based sensors exhibit dynamic, time-varying, hysteretic behaviors on account of their composition. For example, soft piezoresistive sensors show time-varying behaviors due to the dynamic nature of percolated conduction paths during deformation *(21, 23)*. Our cyclic compression characterization experiments demonstrate that the viscoelastic behavior of our printing resins are responsible for any nonlinear behavior from the fluidic sensors. Otherwise, the fluidic sensing approach bypasses other time-varying responses, providing a reliable sensing approach compared to other soft matter-based sensing strategies.

Prediction Results for sHSA Soft Robotic Platform

The results of the hyperparameter search are summarized in Table S3 and Figure S17. The model with the lowest validation loss has a quaternion weight of 100, an initial learning rate of 0.001, 3 LSTM layers and 200 hidden and cell states. This model has a total of 856,007 trainable parameters and retraining it for 1000 epochs takes four hours on a single-GPU (Tesla P100, Nvidia Corporation). Its predictions on the test data exhibit an average position error of 3.2 mm and an average orientation error of 3.6 degrees. Considering the rest length of the sHSA platform of 120

mm and the range of motion in the recordings spanning 39.2 mm and 50.2 degrees, these errors are small. As in Figure 5D, position and orientation errors are plotted over time for test sequences representative of good predictions (Fig. S19A,B) and test sequences representative of worse predictions (Fig. S19C,D). Component-wise predictions of the pose data are plotted against motion capture ground truth for the four aforementioned test sequences (Figs. S20-S23), and for the test sequence shown in Figure 5D (Fig. S24).

The predictions for the test sequences, during which the platform moves continuously without stopping (such as in Fig. S18), are observed to be generally better than those in which the platform is held still for some time (such as Fig. S21-S24). One reason could be that the LSTM model learns from voltage changes, ΔV, rather than from absolute voltage values. This can produce larger errors when the platform is held still and the model happens to provide a wrong prediction for many time steps. This would explain the higher variance in quality of predictions for sequences with long holds (compare Fig. S21, S22, S23, S24). Another indication why this could be true is that even for predictions with larger errors, the timing of a pose change is almost always predicted perfectly. This observation of pose prediction aligning so closely with pose change supports the claim that our fluidic sensors are robust compared to other forms of soft robotic sensors that yield slight time-delays in pose/kinematic predictions *(21)*.

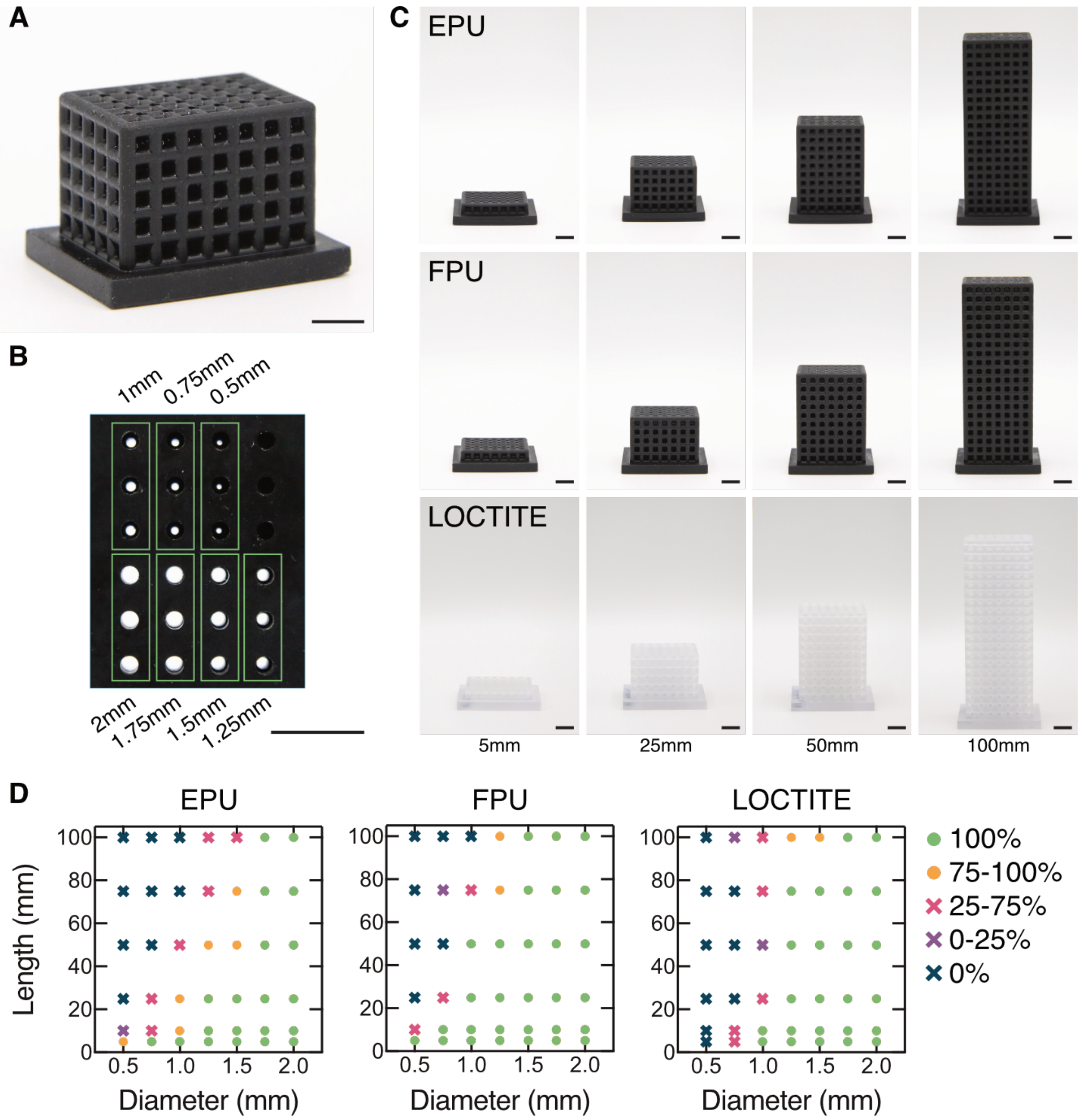

Fig. S1. Fluidic sensor fabricability. (A) A photograph of an example lattice used to characterize fluidic sensor fabricability (EPU, length of 25 mm). (B) Each test lattice has 21 channels embedded along its length. Three channels of varying diameter (0.5 to 2 mm) are clustered as indicated by the green rectangles in the photograph of the underside of a test lattice. The photograph shows open channels in a test lattice with a length of 5mm as viewed from the base, looking down the length of the fluidic channels. (C) Photographs are shown for EPU (top), FPU (middle), and LOCTITE (bottom) test lattices of length 5, 25, 50, and 100 mm. (D) Success of fluidic sensor fabrication (for $n = 12$ sensors per geometry) is provided for EPU (left), FPU (middle), and LOCTITE (right) test lattices for the various channel lengths and diameters screened. (Scale bars represent 10 mm.)

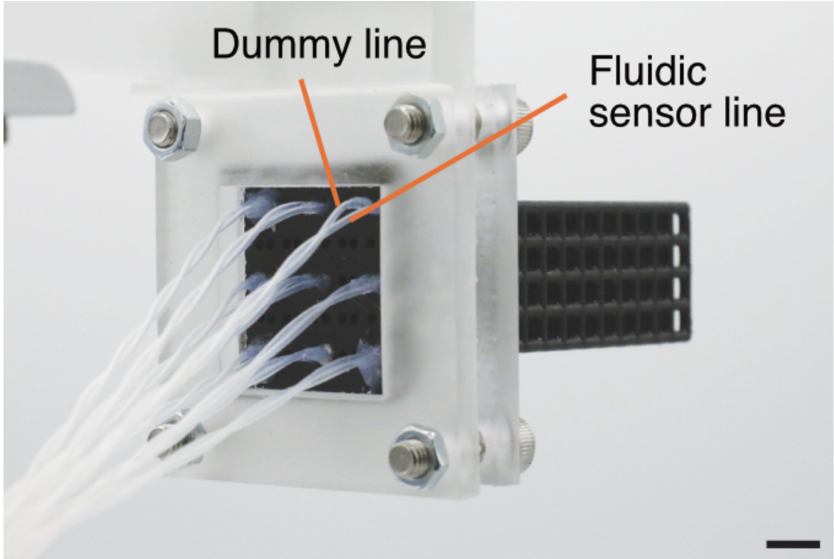

Fig. S2. Mitigating thermal drift with dummy lines. The photograph indicates the fluidic sensor line and dummy line for the top left sensor of the cubic lattice. All nine sensors show the braid-like wrapping of dummy lines around the main tubing connecting the fluidic sensor with the differential pressure sensor. (Scale bar represents 10 mm.)

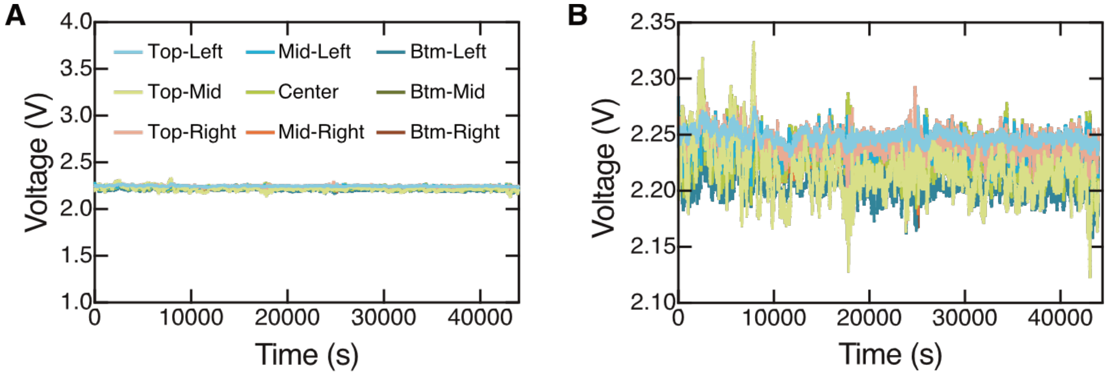

Fig. S3. Thermal drift of sensors. (A) The voltages for the nine sensors of the cubic lattice are shown over a 12-hour time period and plotted over the sensing range of the differential pressure sensor (1 to 4 V). (B) A closer view of the data reveals that sensors generally remain stable over the 12-hour period.

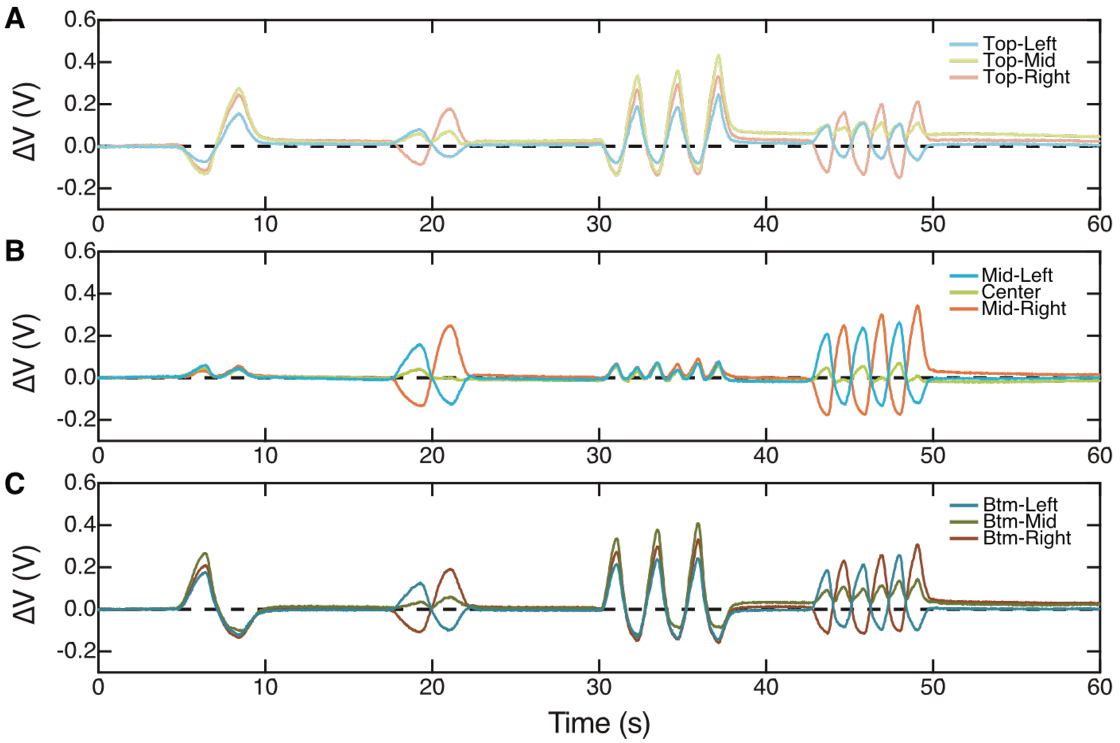

Fig. S4. Detail of sensor response during cubic lattice bending. Detailed plot of voltage change, ΔV, over time from all sensors during the manual bending experiment shown in SI Video 2 and Figure 1F separated into responses for the three (A) Top, (B) Middle (Mid), and (C) Bottom (Btm) fluidic sensors.

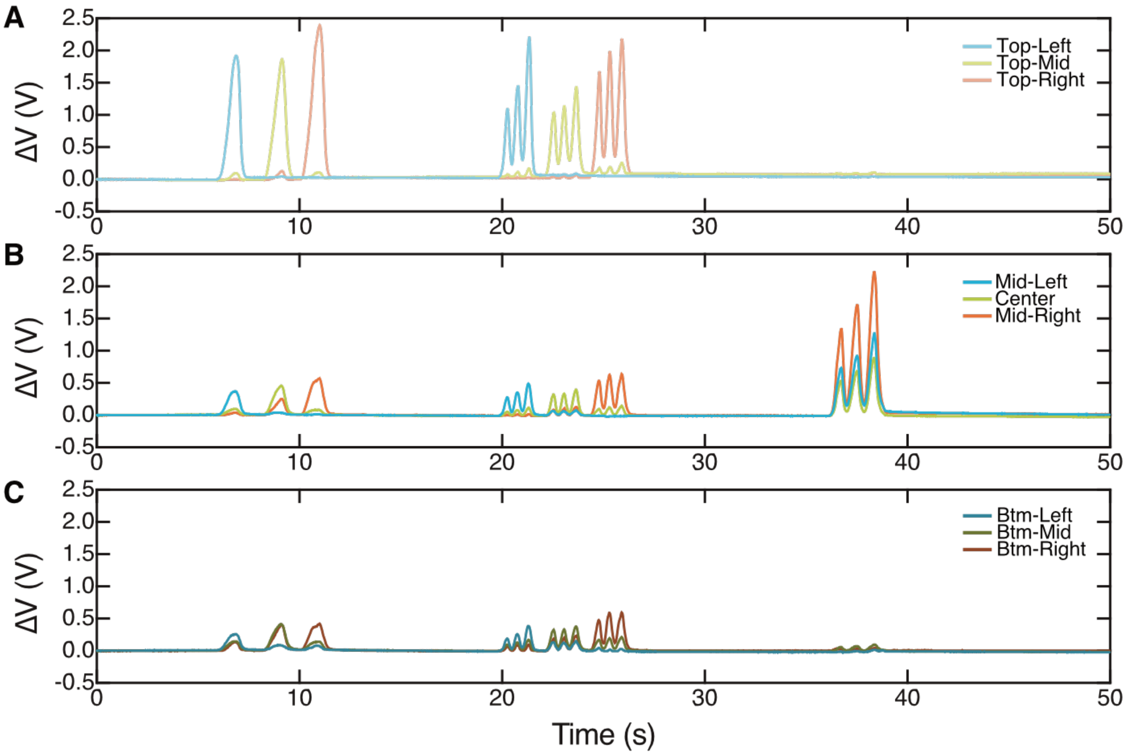

Fig. S5. Detail of sensor response during cubic lattice pressing. Detailed plot of voltage change, ΔV , over time from all sensors during the manual tactile, pressing experiment shown in SI Video 2 and Figure 1H separated into responses for the three (A) Top, (B) Middle (Mid), and (C) Bottom (Btm) fluidic sensors.

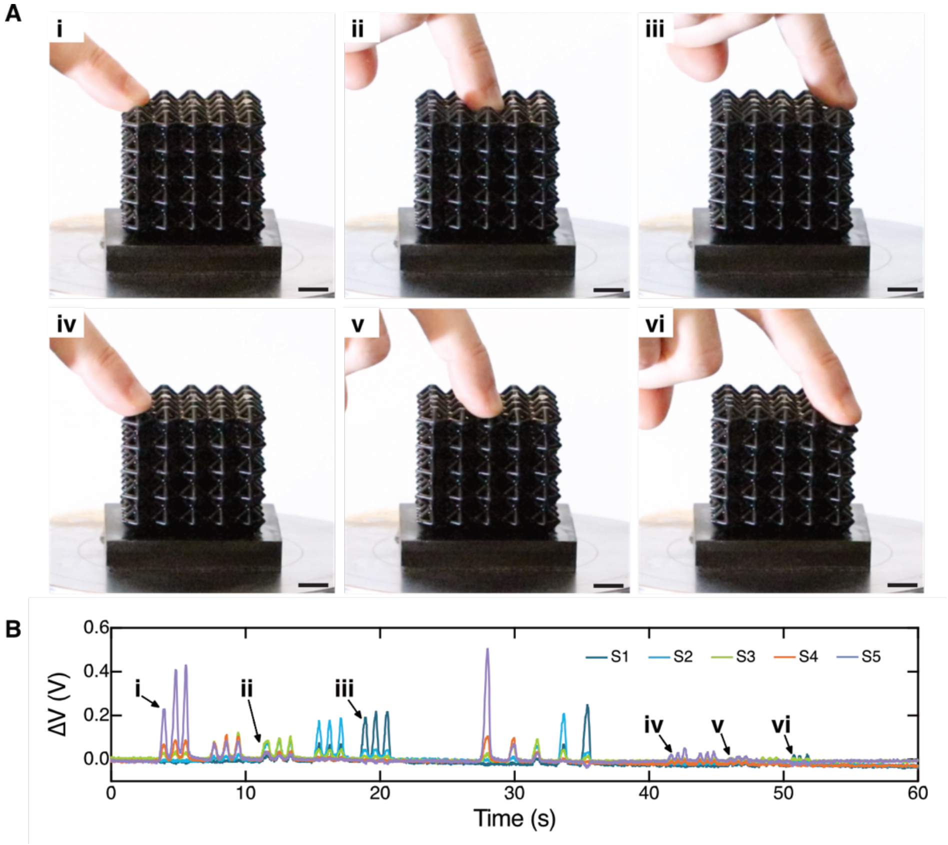

Fig. S6. Sensor response during tactile interactions with the sensorized octahedral lattice. Tops of the sensorized lattice were manually pressed in the following sequence: 3x presses on each fluidic sensor top, starting from S5 to S1; one press on each sensor top in the same order; and 3x presses on the tops of the lattice edge, representing near but off-target locations from the sensors. (A) Photographs and (B) a plot of voltage change, ΔV , over time for all fluidic sensors, S1 through S5, in the octahedral lattice are provided during this tactile interaction sequence, which is also shown in SI Video S3. The photographs in (A) show direct pressing of the tops of sensors (i) S5, (ii) S3, and (iii) S1 three times, as well as (iv-vi) pressing the lattice edge which is not innervated with fluidic sensors. Each interaction is indicated in (B) with an arrow and corresponding label. Scale bar is approximately 1 cm.

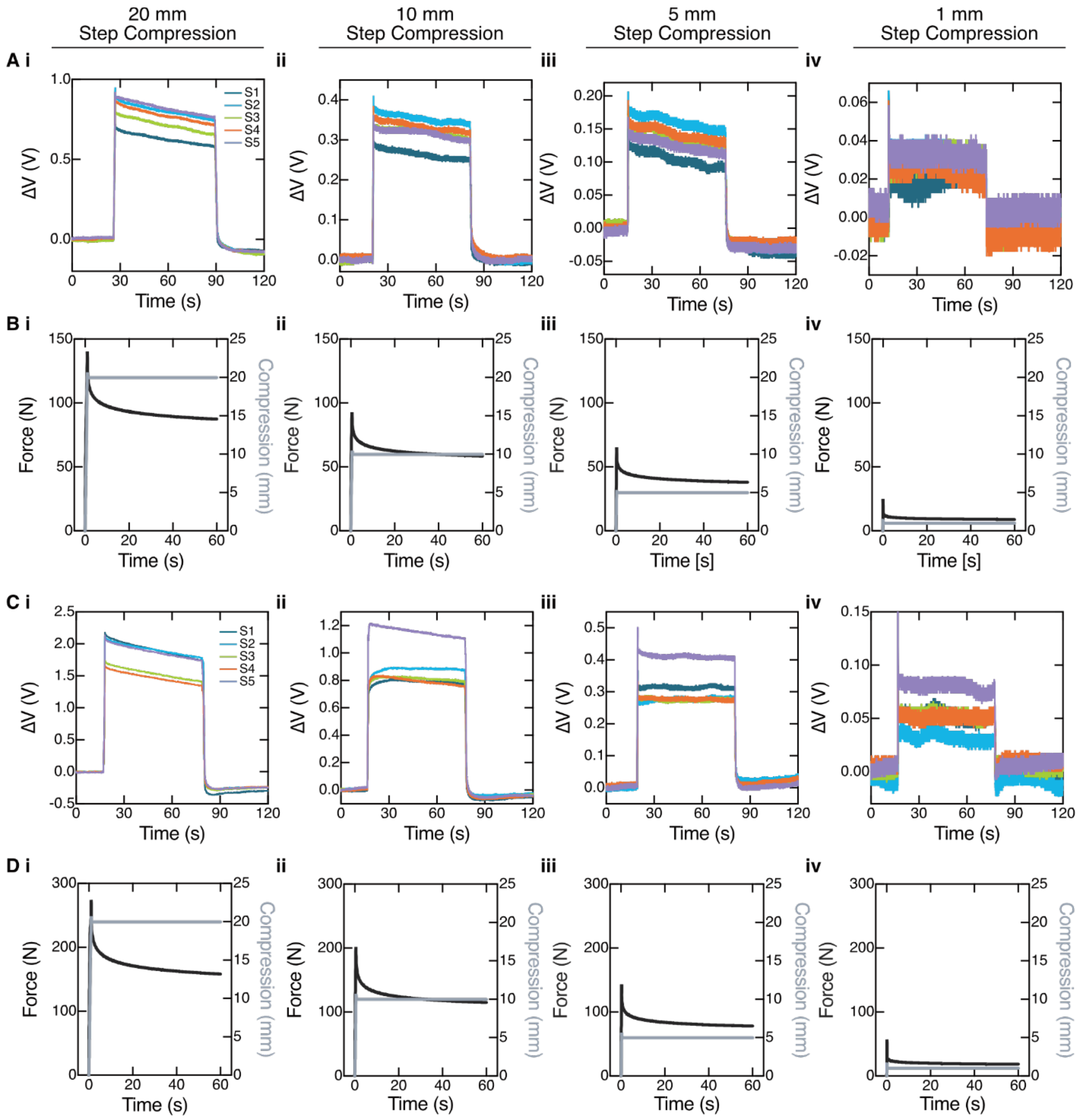

Fig. S7. Sensor responses during step compressions of BCC and octahedral lattices. (A, B) Sensorized BCC and **(C, D)** octahedral lattices undergo step compressions of (**i**) 20, (**ii**) 10, (**iii**) 5, and (**iv**) 1 mm for 60 sec. Sensor responses from the five fluidic sensors S1-S5 are shown in (**A**) and (**C**) for the BCC and octahedral lattices, respectively, as a function of voltage change, ΔV, over time. Sensor readings before and after the step compression show initial and recovering response after compressive load is removed. (**B**) and (**D**) provide the corresponding force and compression over time measured in the BCC and octahedral lattices, respectively, during the 60 sec hold. Plots in **(A,C)** do not maintain identical ΔV scaling to show that sensor response

corresponds with stress relaxation. Data for 20 and 1 mm compressions with equivalent y-axis scales are shown in Figure 3.

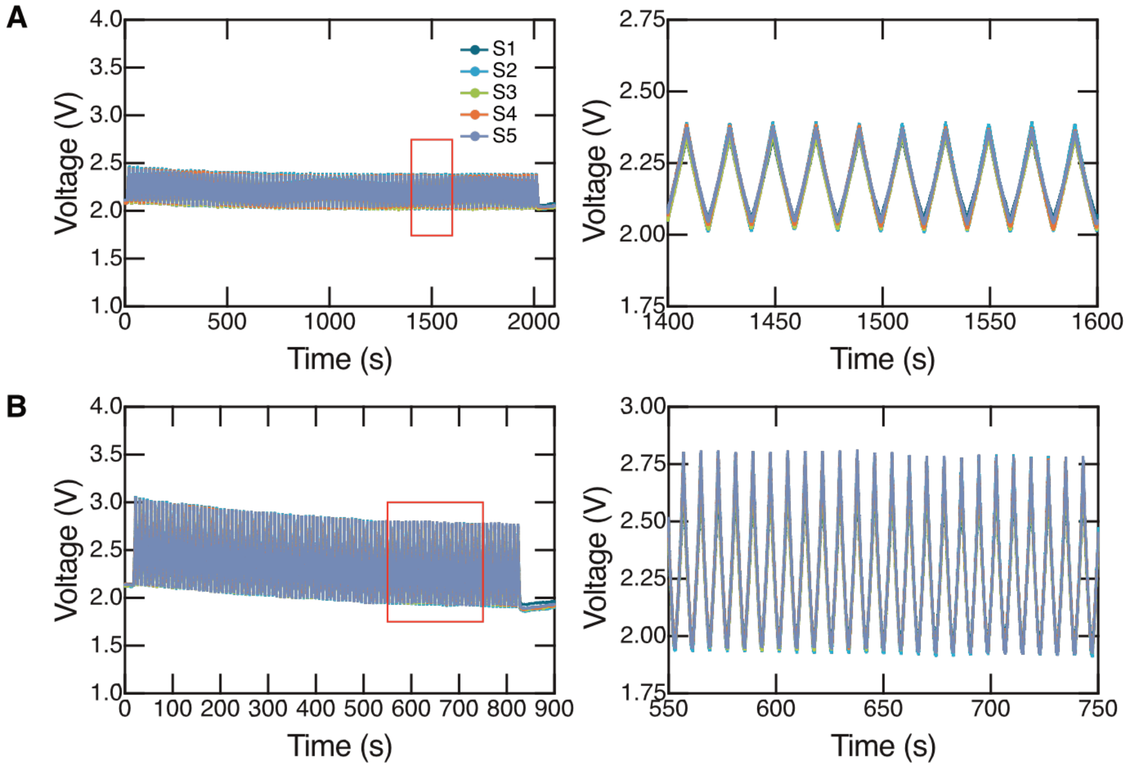

Fig. S8. Cyclic compression of sensorized BCC lattice. Plots of voltage versus time for the five fluidic sensors of the sensorized BCC lattice (sensors S1 through S5 of Figure 2A) during 100 cyclic compressions (A) to 10mm at 1 mm/s and (B) to 20mm at 5 mm/s. Plots in the right column are insets of left column plots indicated by the red rectangle.

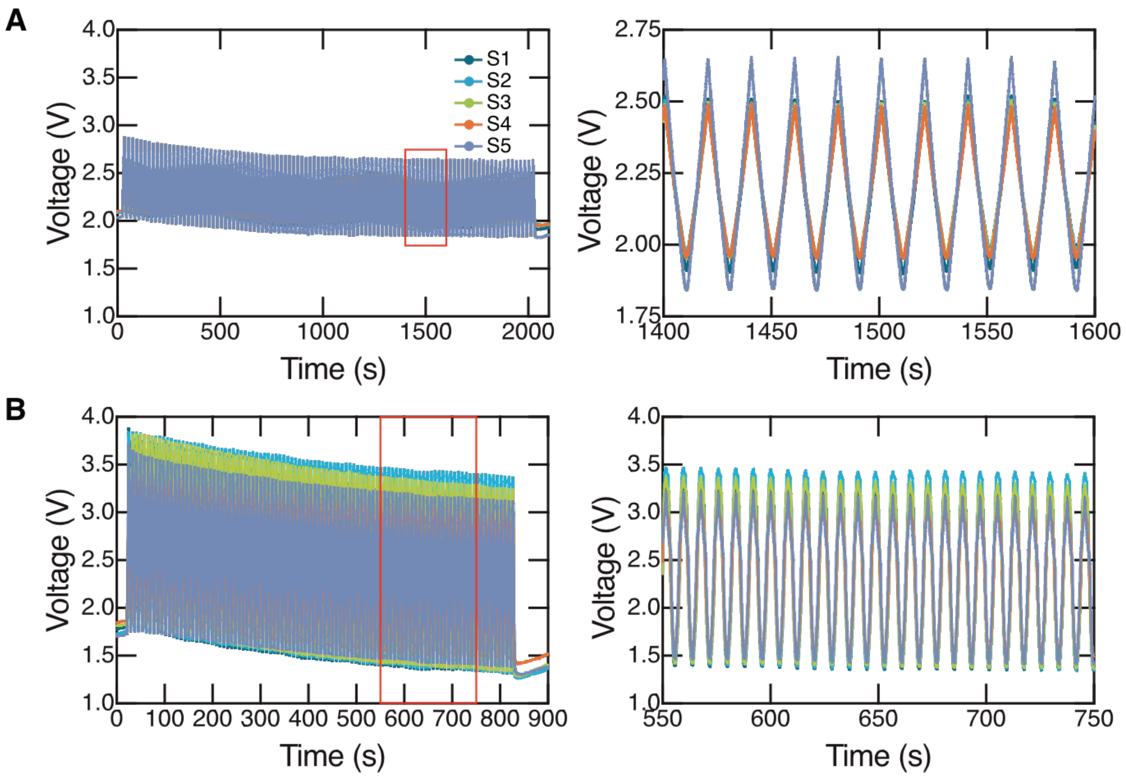

Fig. S9. Cyclic compression of sensorized octahedral lattices. Plots of voltage versus time for the five fluidic sensors of the sensorized octahedral lattice (sensors S1 through S5 of Figure 2B) during 100 cyclic compressions (A) to 10mm at 1 mm/s and (B) to 20mm at 5 mm/s. Plots in the right column are insets of left column plots indicated by the red rectangle.

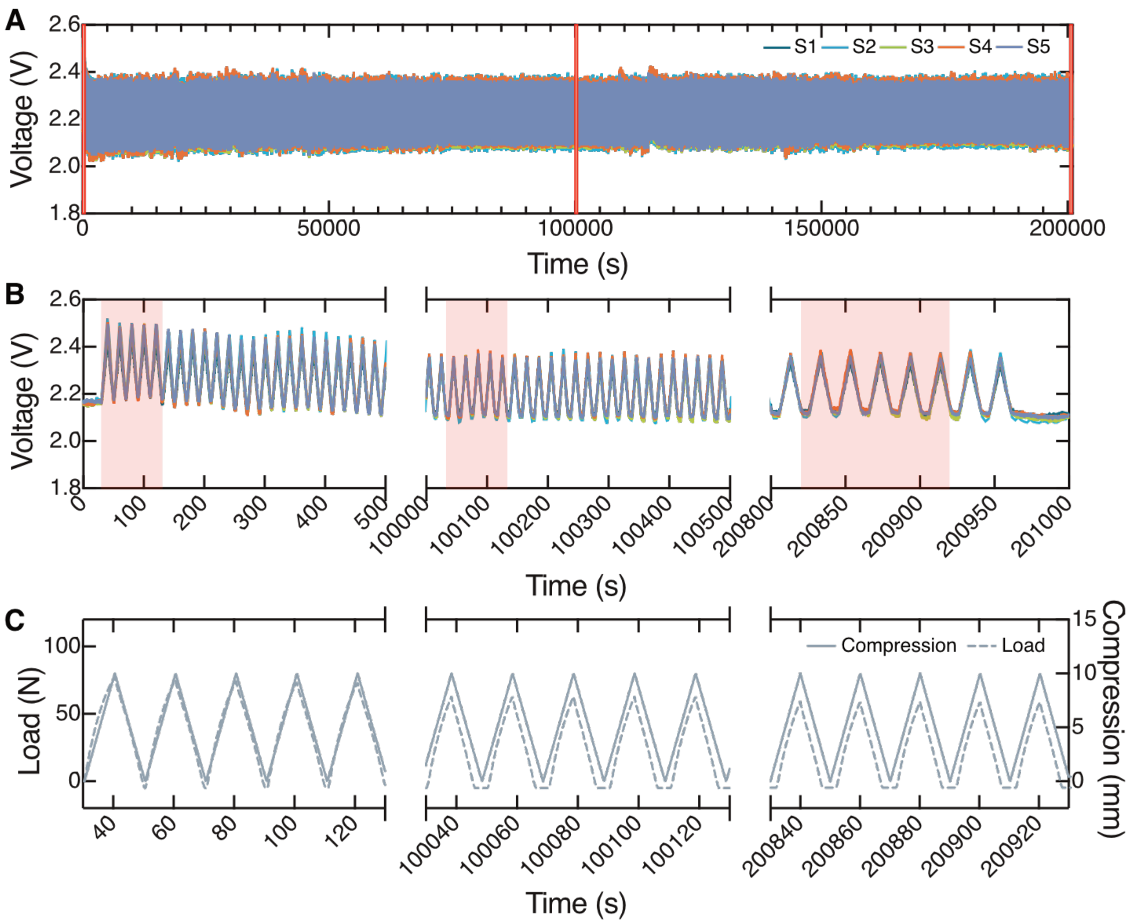

Fig. S10. Sensor characterization during 10,000 cycles of compression. (A) A plot of voltage versus time for the five sensors of the sensorized BCC lattice (sensors S1 through S5 of Figure 2A) during 10,000 cycles of compression to 10mm at 1 mm/s. (B) The three voltage versus time plots correspond to the three insets marked by the three red rectangles in (A). (C) Plots of load (dashed line) and compression (solid line) versus time for the three regions of (B) marked by the shaded red rectangles.

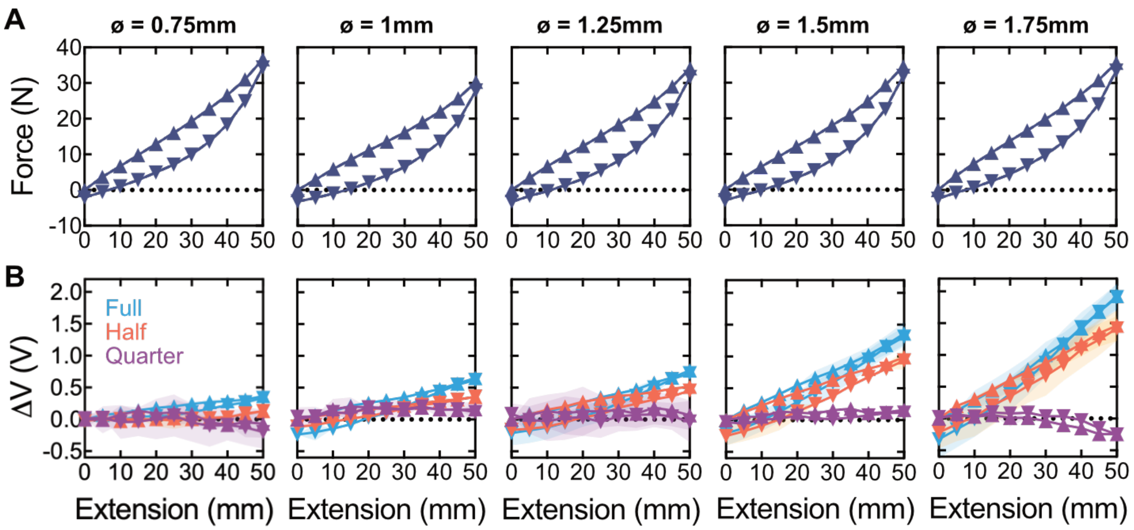

Fig. S11. Characterization of sHSA Full, Half, and Quarter Sensors of Varying Diameter. (A) Plots of extension force and (B) voltage change, ΔV, for Full, Half, and Quarter Sensors versus extension are provided. Columns of plots correspond to data for sHSAs with Full, Half, and Quarter sensors with diameters of 0.75mm, 1mm, 1.25mm, 1.5mm, and 1.75mm. Error bands represent standard deviation ($n = 3$). Triangles pointing upwards and downwards represent data points during extension from 0 to 50mm and from 50 to 0mm, respectively.

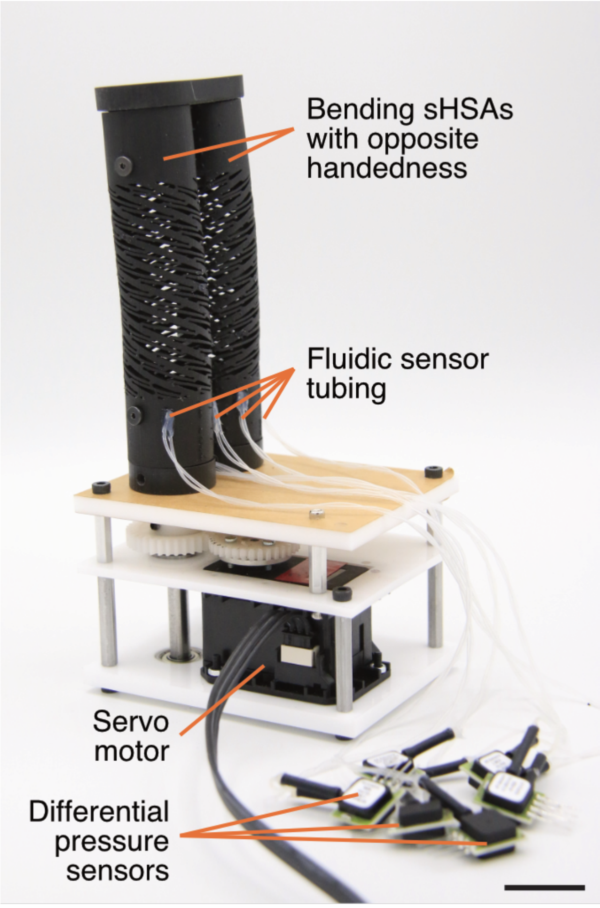

Fig. S12. sHSA Bending Actuator. Photograph of the sHSA bending actuator comprised of a 2x1 grid of sHSAs with opposite handedness. Each sHSA has 1.0mm diameter ``3/4", ``1/2", and ``1/4" sensors. Scale bar represents approximately 25 mm.

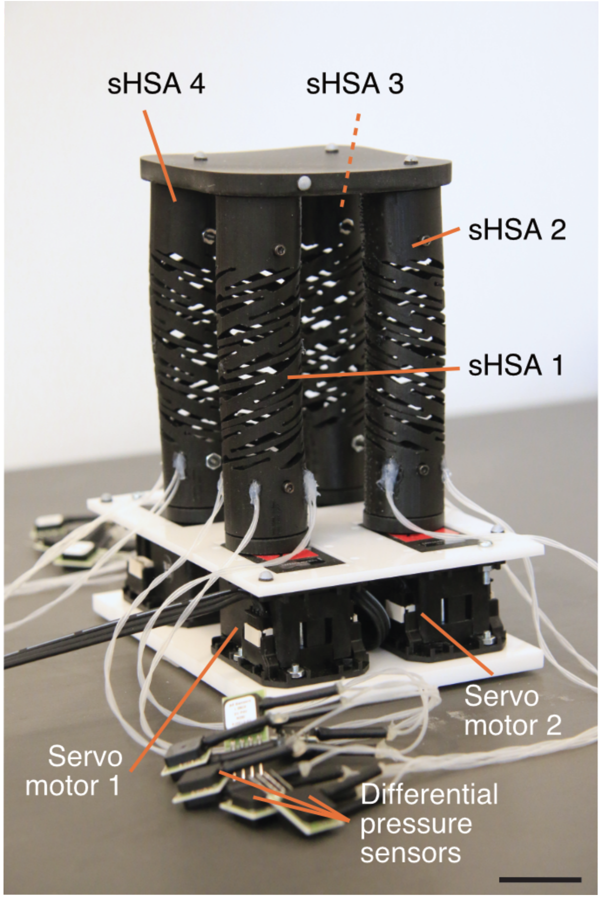

Fig. S13. sHSA Platform. Photograph of the sHSA platform comprised of a 2x2 grid of sHSAs with alternating handedness. Each sHSA has 1.5mm diameter ``Full'', ``Half'', and ``Quarter'' sensors. Scale bar represents approximately 25 mm.

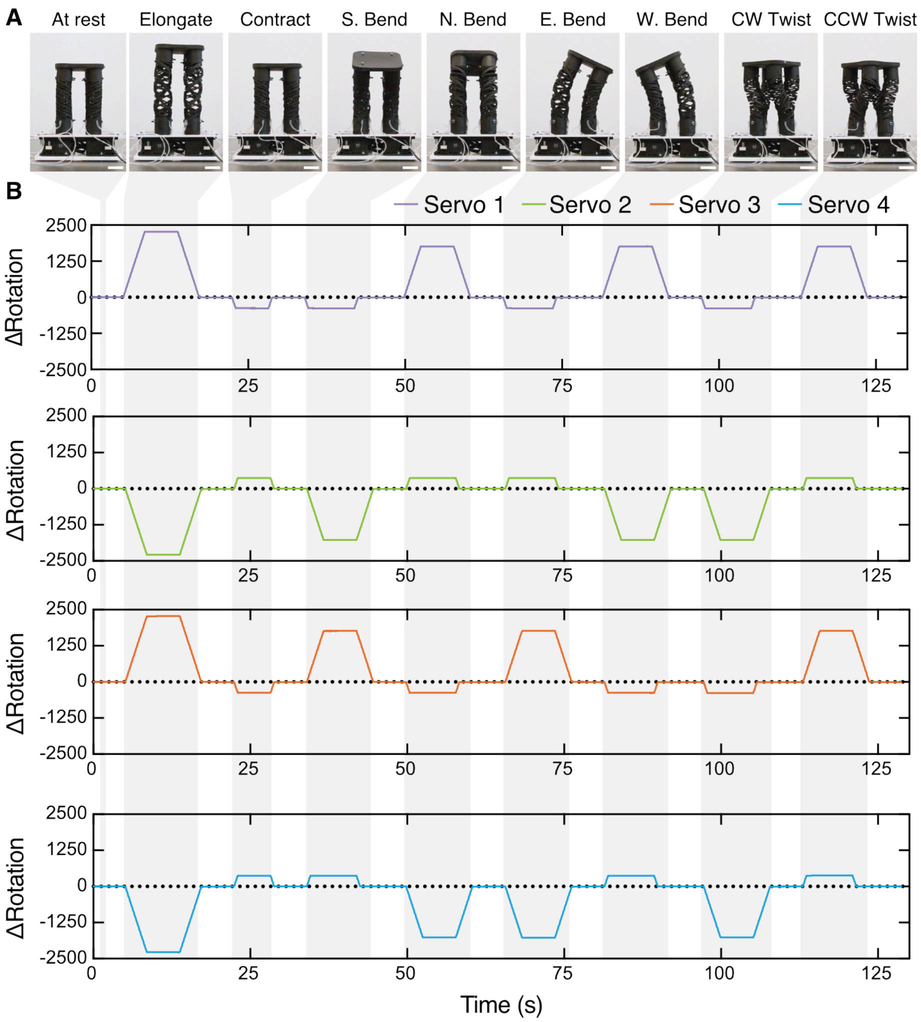

Fig. S14. Servo rotations for sHSA platform. The data corresponds to Figure 5A-B in the main manuscript, with (A) photographs of the same platform poses provided. (B) The corresponding change of servo rotation (ΔRotation) for Servos 1 through 4, which drive sHSAs 1 through 4, respectively, are provided as a function of time. Scale bars represent 25 mm.

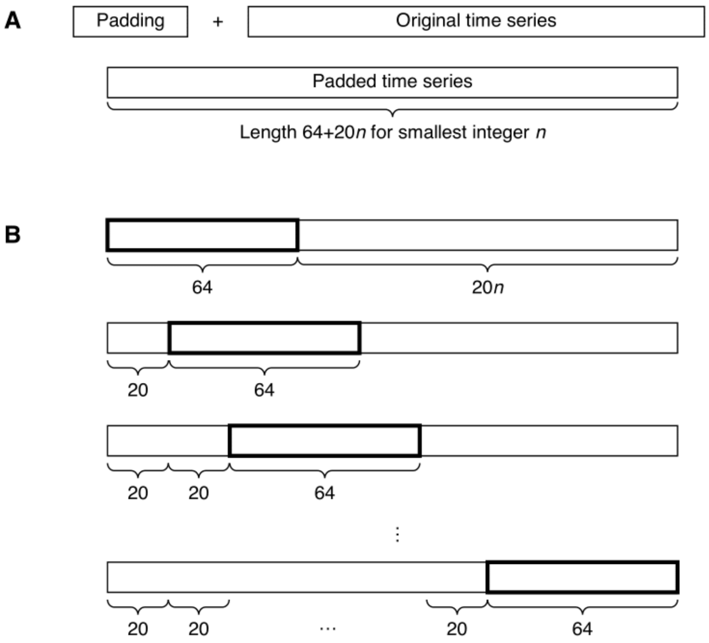

Fig. S15. Preprocessing of training data. Sequences of length 64 are extracted from each of the 180 experiments. (A) Each of the original time series is padded such that the new length equals $64 + 20n$ for the smallest integer n. The data with which the time series is padded is the first data point of that sequence, which consists of zero pressures for the inputs and the initial pose for the outputs. (B) Extracting n data sequences of length 64 by sliding a window (marked in bold) over the padded time series with a stride of 20. This method simultaneous augments the data as four consecutive length-64 sequences contain overlapping time points.

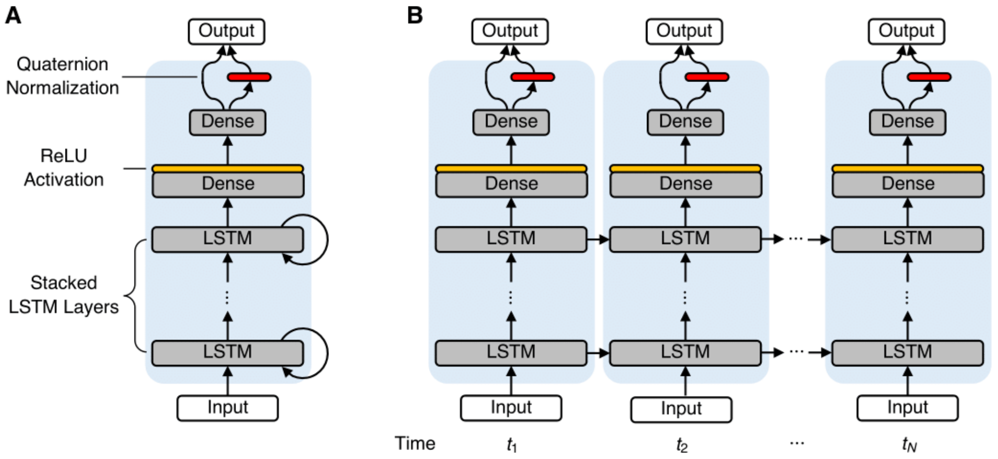

Fig. S16. LSTM for proprioceptive sensing in sHSA platform. (A) Model architecture. The 12-dimensional input is passed through stacked LSTM layers, where number of layers and size of their hidden and cell states are tunable hyperparameters. A dense layer with equal size is followed by ReLU activation and another dense layer that outputs a 7-dimensional vector. This vector is passed through a layer that normalizes the four values corresponding to the quaternion outputs. Dropout with probability 0.2 is applied to the first dense layer and each LSTM layer (not shown). (B) Forward pass unrolled through time for input sequence of length N.

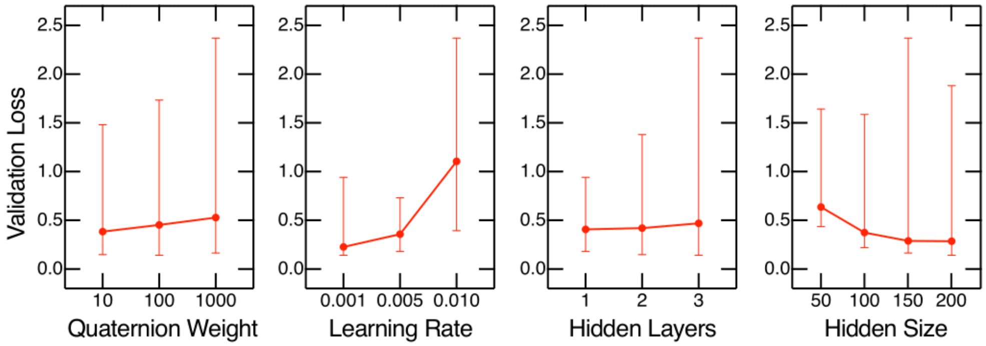

Fig. S17. Hyperparameter Search Results. A grid search is performed over all combinations of the displayed values for each hyperparameter. The validation loss is averaged over three runs for each combination to account for randomness. The plots show median validation loss and range (error bars) of the marginal distribution over all other hyperparameters. See Tab. S1 for numerical values and further explanation.

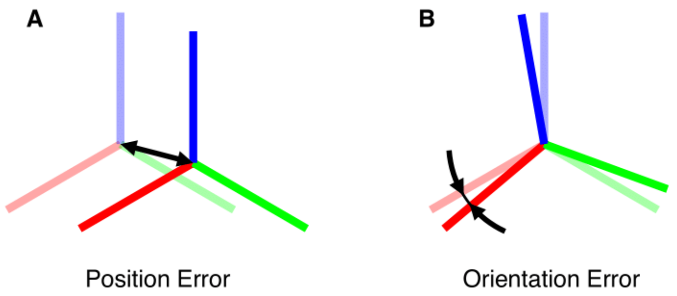

Fig. S18. Error Metrics. Schematic to illustrate the error metrics that are used to evaluate the final model performance on the test data. (A) The position error is defined as the distance between the origins of the predicted pose (opaque RGB coordinate axes) and the ground truth pose (transparent RGB coordinate axes). (B) The orientation error is defined as the relative rotation between the poses.

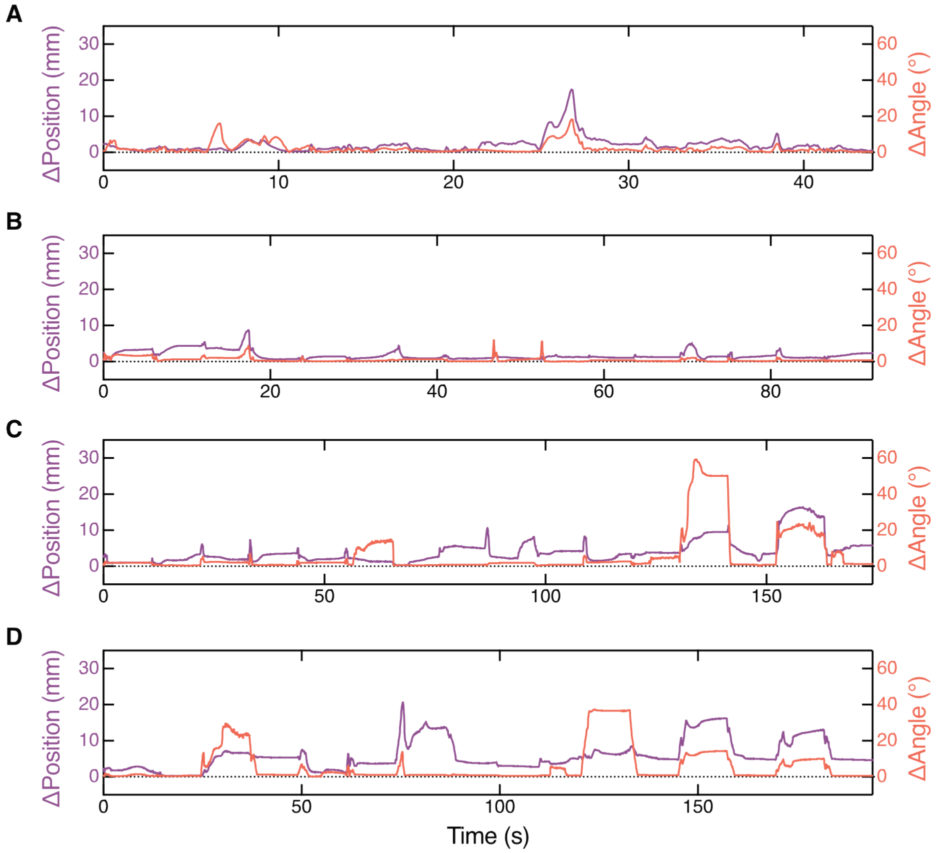

Fig. S19. Representative Prediction Errors. Position (ΔPosition) and orientation (ΔAngle) error over time for a set of representative test sequences giving better (A,B) and worse (C,D) predictions. (A) Test sequence 1 has an average position error of 2.1 mm and an average orientation error of 2.3 degrees. (B) Test sequence 2 has an average position error of 1.8 mm and an average orientation error of 1.0 degrees. (C) Test sequence 3 has an average position error of 4.1 mm and an average orientation error of 6.3 degrees. (D) Test sequence 4 has an average position error of 5.9 mm and an average orientation error of 5.9 degrees.

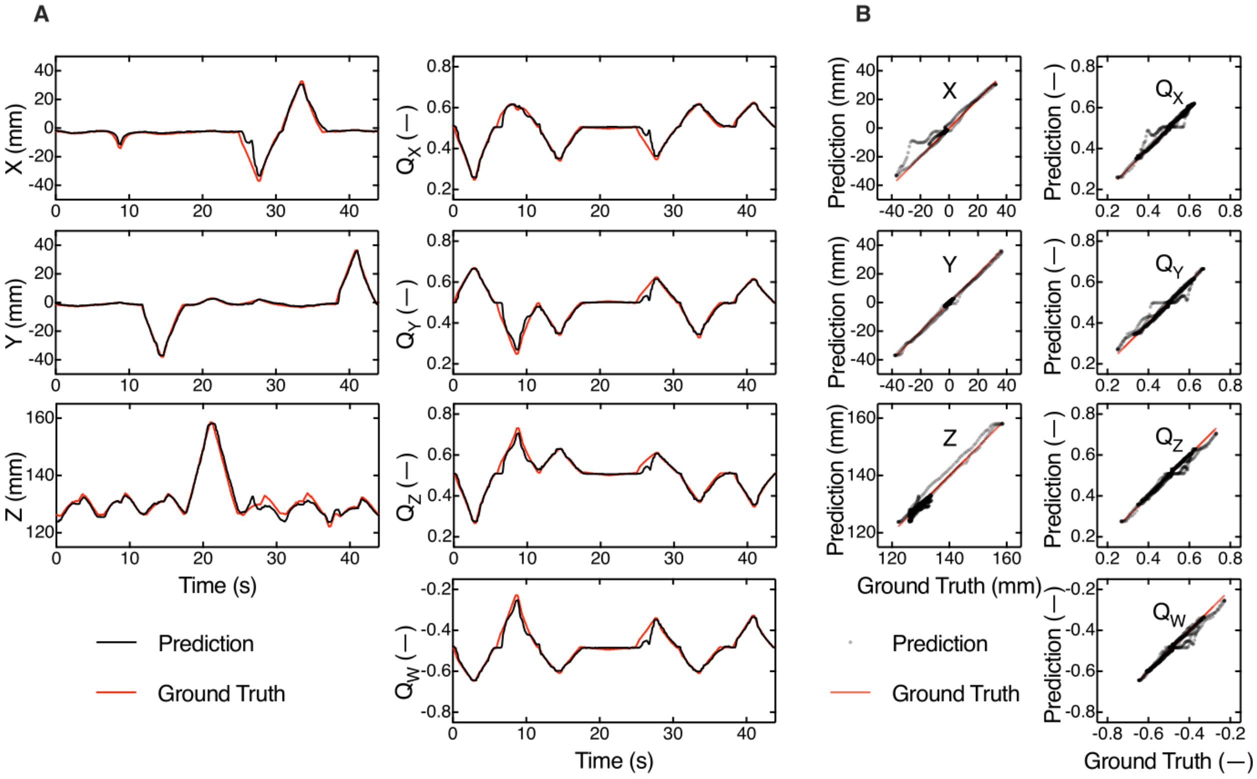

Fig. S20. Component-wise predictions for test sequence 1. Prediction and motion capture ground truth for a test sequence that is representative of a typical good prediction, broken down into position (X,Y,Z) and quaternion (Q_X, Q_Y, Q_Z, Q_W) components. (A) Prediction (black) and ground truth (red) over time. (B) Prediction (grey dots) over ground truth (red line).

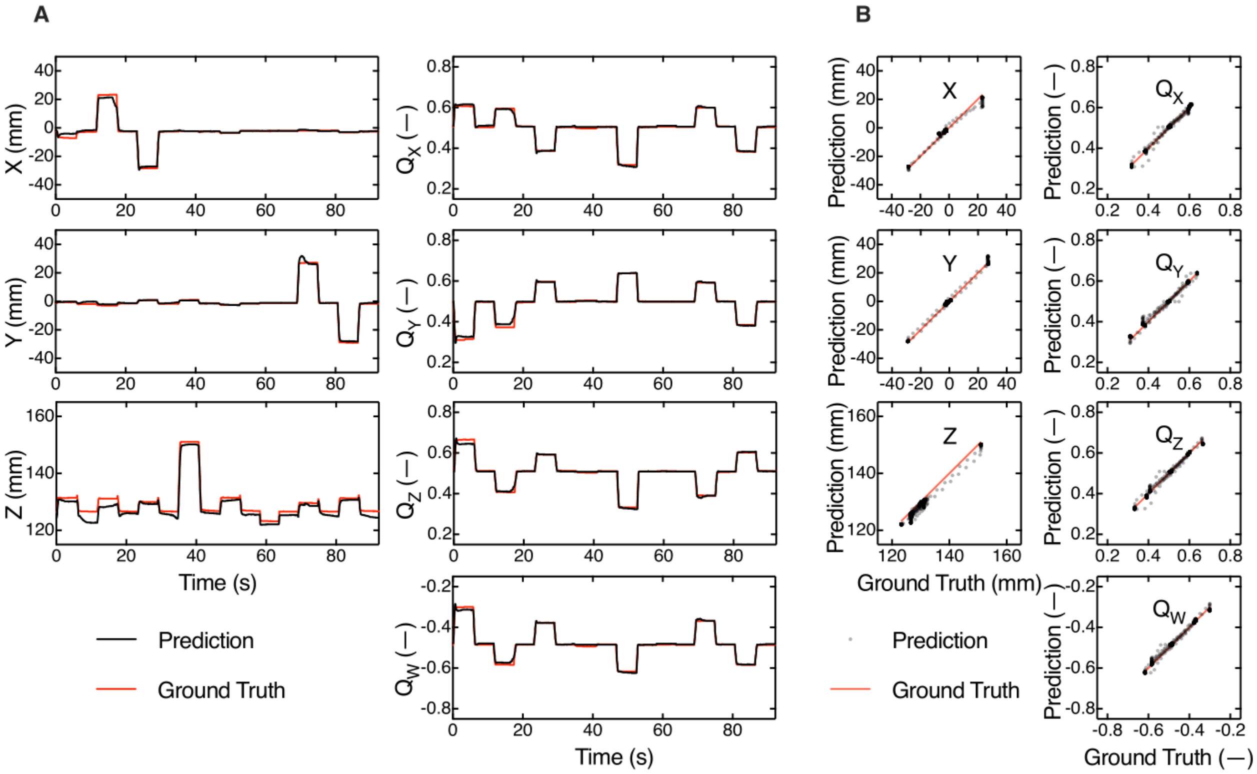

Fig. S21. Component-wise predictions for test sequence 2. Prediction and motion capture ground truth for a test sequence that is representative of a typical good prediction, broken down into position (X,Y,Z) and quaternion (Q_X, Q_Y, Q_Z, Q_W) components. (A) Prediction (black) and ground truth (red) over time. (B) Prediction (grey dots) over ground truth (red line).

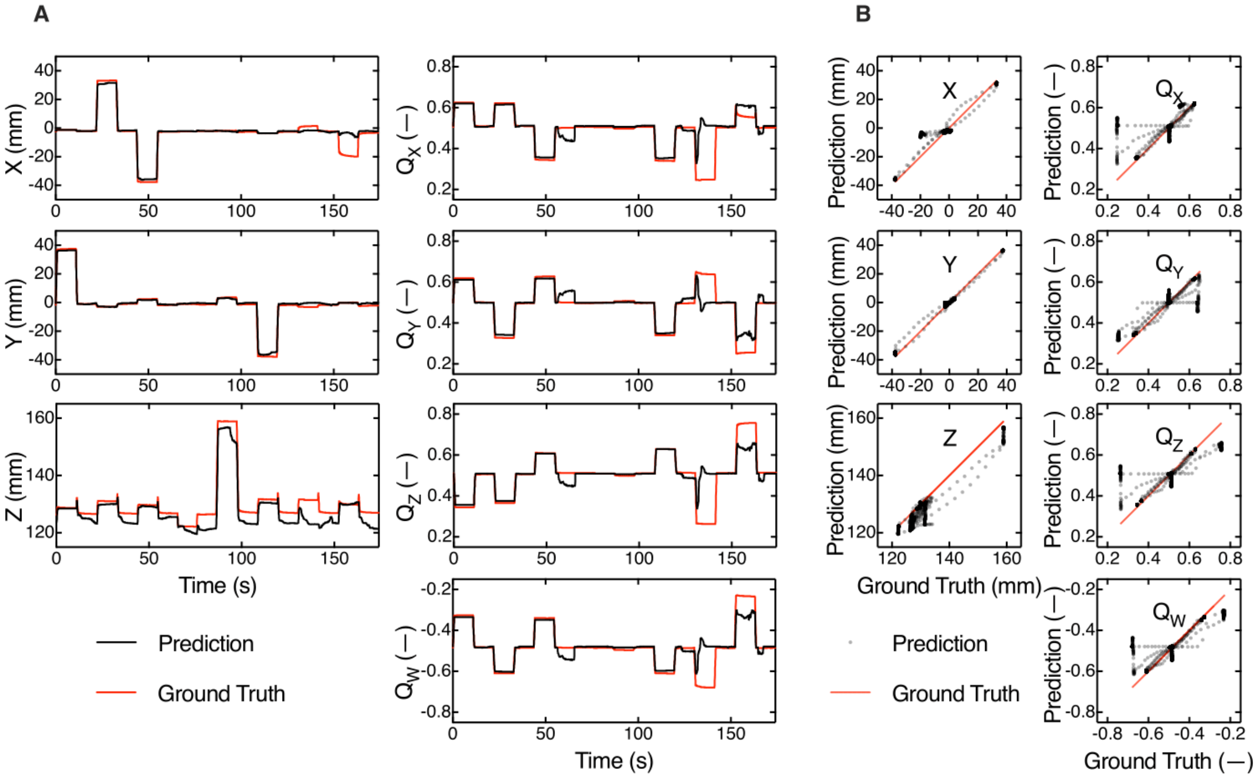

Fig. S22. Component-wise predictions for test sequence 3. Prediction and motion capture ground truth for a test sequence that is representative of a typical poor prediction, broken down into position (X,Y,Z) and quaternion (Q_X, Q_Y, Q_Z, Q_W) components. (A) Prediction (black) and ground truth (red) over time. (B) Prediction (grey dots) over ground truth (red line).

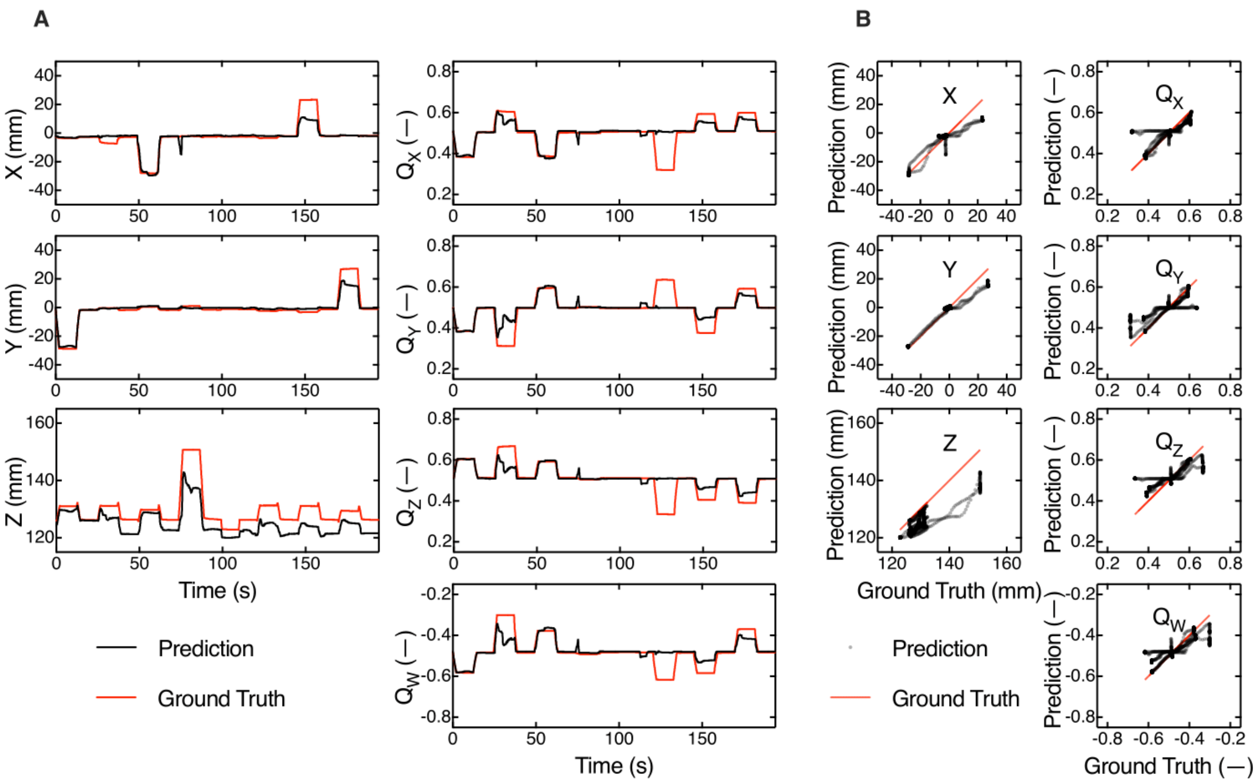

Fig. S23. Component-wise predictions for test sequence 4. Prediction and motion capture ground truth for a test sequence that is representative of a typical poor prediction, broken down into position (X,Y,Z) and quaternion (Q_X, Q_Y, Q_Z, Q_W) components. (A) Prediction (black) and ground truth (red) over time. (B) Prediction (grey dots) over ground truth (red line).

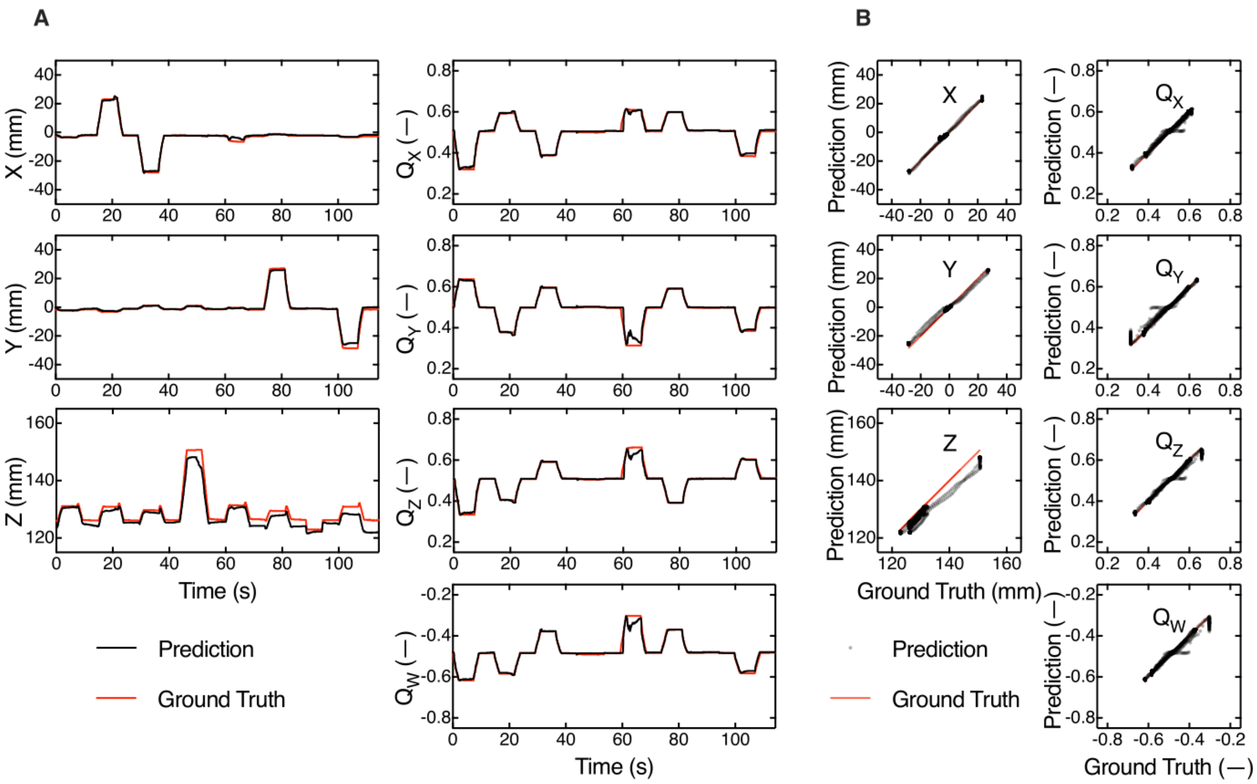

Fig. S24. Component-wise predictions for test sequence shown in Figure 5. Prediction and motion capture ground truth for the test sequence shown in Figure 5, broken down into position (X,Y,Z) and quaternion (Q_X, Q_Y, Q_Z, Q_W) components. (A) Prediction (black) and ground truth (red) over time. (B) Prediction (grey dots) over ground truth (red line).

Lattice (Unit Cell Size)	# Fluidic Sensors	Fluidic Sensor Diameter (mm)	Strut Diameter (mm)	Volume per Fluidic Sensor (mm^3)
Cubic ($5x5x5\ mm^3$)	9	1.25	2	93
Body Centered Cubic, BCC ($10x10x10\ mm^3$)	5	1	2	120
Octahedral ($10x10x10\ mm^3$)	5	1	2	117

Table S1. Sensorized Lattice Design Parameters. All values reported are those used in Rhino 6 to create the sensorized lattices. The fluidic sensor volumes (i.e., the volume innervating the lattice plus the volumes routing to ports for external tubing) are measured using the Grasshopper command "Volume." All pressure changes in the sensorized lattices are measured with a 0-5 in H_20 differential pressure sensor.

sHSA Design, (Fluidic Sensor Diameter)	Fluidic Sensor ID	Sensor Length (mm)	Fluidic Sensor Volume (mm^3)	Differential Pressure Sensor Range (in H_2O)
Straight (0.75 mm)	Full	139	62	0 – 1
	Half	73	32	0 – 1
	Quarter	41	18	0 – 0.05
Straight (1 mm)	Full	139	109	0 – 1
	Half	73	57	0 – 1
	Quarter	41	32	0 – 0.05
Straight (1.25 mm)	Full	139	171	0 – 1
	Half	73	89	0 – 1
	Quarter	41	51	0 – 0.05
Straight (1.5 mm)	Full	139	246	0 – 1
	Half	73	128	0 – 1
	Quarter	41	73	0 – 0.05
Straight (1.75 mm)	Full	139	335	0 – 1
	Half	73	175	0 – 1
	Quarter	41	99	0 – 0.05
Bending (1 mm)	3/4	114	90	0 – 1
	1/2	71	56	0 – 1
	1/4	51	40	0 – 0.05

Table S2. Sensorized HSA (sHSA) Design Parameters. All values reported are those used in Rhino 6 to create the sHSAs. The fluidic sensor lengths and volumes (i.e., the volume innervating the HSA structure plus the volumes routing to ports for external tubing) are measured using the Grasshopper commands “Length” and “Volume,” respectively.

Hyperparameter	Value	Minimum	Median	Maximum
Quaternion Weight	10	0.1498	0.3837	1.4822
	100	**0.1440**	0.4529	1.7351
	1000	0.1649	0.5299	2.3716
Learning Rate	0.001	**0.1440**	0.2280	0.9411
	0.005	0.1809	0.3571	0.7338
	0.01	0.3934	1.1064	2.3716
Hidden Layers	1	0.1809	0.4093	0.9411
	2	0.1505	0.4210	1.3825
	3	**0.1440**	0.4713	2.3716
Hidden Size	50	0.4368	0.6357	1.6430
	100	0.2211	0.3740	1.5879
	150	0.1661	0.2908	2.3716
	200	**0.1440**	0.2882	1.8824

Table S3. Hyperparameter search results. A grid search is performed over all combinations of values shown for each hyperparameter in the Value column. For each specific combination of hyperparameters, the validation loss averaged over three trials is recorded. For a certain value of one hyperparameter, the marginal distribution of validation losses is computed over all values of all other hyperparameters. Minimum, median, and maximum of this marginal distribution are tabulated. I.e., the first row means all 36 possible hyperweight combinations with a quaternion weight of 10 result in a median validation loss of 0.3837. The minimal validation loss for each hyperparameter is shown in bold.

Movie S1.

Evacuation of innervating fluidic sensor networks. Aspiration of excess, non-polymerized resin from fluidic sensor networks is shown with a cubic lattice printed from transparent resin.

Movie S2.

Manual bending and touch interactions with a sensorized, cubic lattice beam. Voltage changes from nine fluidic sensors in a sensorized cubic lattice are shown over time at 2x playback.

Movie S3.

Tactile interactions with a sensorized octahedral lattice. Voltage changes from five fluidic sensors in a sensorized octahedral lattice are shown over time at 2x playback.

Movie S4.

Step Compression of Octahedral Lattice, 20 mm for 60 sec. The sensorized octahedral lattice undergoes a step compression of 20 mm for 60 sec, revealing the stress relaxation and shape recovery of the lattice after deformation.

Movie S5.

Sensor responses during sHSA finger actuation. Voltage changes from six fluidic sensors in a bending sHSA finger are shown over time at 4x playback.

Movie S6.

Sensor response from multi-DOF sHSA segment. Voltage changes from 12 fluidic sensors are shown over time at 4x playback.

Movie S7.

Pose prediction with sHSA Platform: Example 1. Animations of predicted and ground truth poses as shown in Figure 5c are provided alongside plots of position and rotational angle error from Figure 5d. Playback is 4x.

Movie S8.

Pose predictions with sHSA Platform: four representative sequences. Animations of predicted and ground truth poses for four test sequences are provided alongside corresponding plots of position and rotational angle error from Figure S17. Playback is 4x.
